# Comparative Analysis of Type IV Pilin in *Desulfuromonadales*

**DOI:** 10.3389/fmicb.2016.02080

**Published:** 2016-12-21

**Authors:** Chuanjun Shu, Ke Xiao, Qin Yan, Xiao Sun

**Affiliations:** State Key Laboratory of Bioelectronics, School of Biological Science and Medical Engineering, Southeast UniversityNanjing, China

**Keywords:** conductive pilin, extracellular electron transfer, structure, phylogenetic analysis, gene fission

## Abstract

During anaerobic respiration, the bacteria *Geobacter sulfurreducens* can transfer electrons to extracellular electron accepters through its pilus. *G. sulfurreducens* pili have been reported to have metallic-like conductivity that is similar to doped organic semiconductors. To study the characteristics and origin of conductive pilin proteins found in the pilus structure, their genetic, structural, and phylogenetic properties were analyzed. The genetic relationships, and conserved structures and sequences that were obtained were used to predict the evolution of the pilins. Homologous genes that encode conductive pilin were found using PilFind and Cluster. Sequence characteristics and protein tertiary structures were analyzed with MAFFT and QUARK, respectively. The origin of conductive pilins was explored by building a phylogenetic tree. Truncation is a characteristic of conductive pilin. The structures of truncated pilins and their accompanying proteins were found to be similar to the N-terminal and C-terminal ends of full-length pilins respectively. The emergence of the truncated pilins can probably be ascribed to the evolutionary pressure of their extracellular electron transporting function. Genes encoding truncated pilins and proteins similar to the C-terminal of full-length pilins, which contain a group of consecutive anti-parallel beta-sheets, are adjacent in bacterial genomes. According to the genetic, structure, and phylogenetic analyses performed in this study, we inferred that the truncated pilins and their accompanying proteins probably evolved from full-length pilins by gene fission through duplication, degeneration, and separation. These findings provide new insights about the molecular mechanisms involved in long-range electron transport along the conductive pili of *Geobacter* species.

## Introduction

Electricigens are microbes that are capable of extracellular electron transfer and electricity production. These microbes are currently of great interest for their practical applications in global geochemical cycling of metals, minerals, carbon in the environment, bioremediation of contaminants, and microbe-electrode interactions. Additionally, electricigens have potential applications in energy harvesting and the renewable production of organic commodities from carbon dioxide (Lovley et al., 2011; Malvankar and Lovley, 2012, 2014; Lovley and Nevin, 2013). There are three potential mechanisms for electricigens to transfer electrons to extracellular acceptors: (a) electron transfer via soluble electron-shuttling molecules, such as methylviologen (Lovley, 2006); (b) short-range direct electron transfer via redox-active proteins such as c-type cytochromes (Wrighton et al., 2011); and (c) long-rang electron transport via conductive pili, for instance the pili of *Geobacter sulfurreducens* PCA (Reguera et al., 2005).

*G. sulfurreducens* is an electricigen and its pili have been regarded as “microbial nanowires” because of their electrical conductivity, which has been attributed to overlapping pi-pi orbitals (Reguera et al., 2005; Malvankar et al., 2011, 2015; Reardon and Mueller, 2013; Vargas et al., 2013). Because of the environmental and practical significance of extracellular electron transfer along pili and the metallic-like conductivity of *Geobacter* pili, which has never been reported for other Type IV Pili, understanding the detailed mechanism of this special characteristic is very important.

Type IV pili are protein filaments that are built up by small monomers called pilins, in a repetitive and symmetrical way (Craig et al., 2004). Thus, the specific amino acid sequences and structures of monomers will clearly influence the assembly of a pilus and its functions (Vargas et al., 2013; Xiao et al., 2015). Previous researches have suggested that the length of the amino acid sequence and the conformation of *G. sulfurreducens* pilins leads to a compact assembly mode that may account for the electrical conductivity (Malvankar et al., 2011; Xiao et al., 2016). Therefore, analyses of the sequence and structural features of type IV pilins (TFPs) are warranted to provide a better understand of the functions and mechanisms of these proteins.

*Geobacter* pilins are type IVa pilins that are characterized by shorter signal peptides (5–6 amino acids) and shorter mature sequences than type IVb pilins (Craig et al., 2004). Notably, the major pilin PilA of *G. sulfurreducens* is much shorter than “normal” type IVa pilins because the short PilA is encoded by the truncated GSU1496 gene, which does not encode the C-terminal globular domain found in the longer pilins (Reardon and Mueller, 2013). In *Geobacter*, biological experiment structure of pilin is only accessible for GSU1496 (PDB ID: 2M7G), which only contains an alpha-helix. However, normal Type IV pilins have both a complete alpha-helix and a C-terminal globular domain, such as the major pilins of *Neisseria gonorrhoeae* (PDB ID: 1AY2), *Pseudomonas aeruginosa* (PDB ID: 1DZO) and *Vibrio cholera* (PDB ID: 1OQV).

GSU1496 shares an operon with GSU1497, which is located directly downstream of the former (Methe et al., 2003; Holmes et al., 2006). Furthermore, GSU1497 and GSU1496 are functionally related, the mutation that knockout GSU1497 gene was found that may result in no expression of PilA and slower growth of the mutant in microbial fuel cells (Nevin et al., 2009; Richter, 2011). The extreme short length of PilA, the adjacent location of GSU1496 and GSU1497 gene on the *G. sulfurreducens* KN400 genome, and their coexpression, all indicate that the major pilin (PilA) of *G. sulfurreducens* is a truncated version of full-length TFPs.

To further investigate the characteristics of *Geobacter* pili and explore the possible origin of the conductive pili, in this study we performed evolutionary analysis to reveal the relationships between short and normal length pilins mostly from *Proteobacteria*, most of which were electricigens (Logan, 2009). We also examined the general characteristics of the major pilins. These analyses will help to better understand the features of electrically conductive pili, and guide further experiments into the mechanisms involved in extracellular electron transfer along these pili.

## Materials and methods

### Sequence preparation

RefSeq is an open access database that contains an annotated and curated non-redundant collection of sequences representing genomes, transcripts, and proteins (Pruitt et al., 2007, 2012). We used PilFind to find all the potential pilins from bacteria in order *Desulfuromonadales*, which includes many electricigens, in RefSeq (release 70) (Imam et al., 2011). Two files of genomic data were obtained from different download paths. One file contained genome information, such as name, length, and number of the genes in the respective genomes; and the other file contained the complete annotations. In this study, short TFPs with < 100 amino acids (the length of the alpha-helix in full-length pilin) were defined as truncated pilins, among them PilA (GSU1496) from *G. sulfurreducens* was a typical example. The other TFPs were defined as full-length pilins. We downloaded 2774 bacteria and archaea genome sequences, including 11 *Desulfuromonadales* strains that had intact annotations, from ftp://ftp.ncbi.nlm.nih.gov/genomes/Bacteria/all.faa.tar.gz to analyze the prevalence of truncated pilins among *Desulfuromonadales* species and to determine the relationship between the truncated and full-length pilins.

We used the Basic Local Alignment Search Tool (BLAST) in NCBI's non-redundant protein sequences (nr) database to identify pilins that were not in RefSeq. Position-Specific Iterative BLAST (PSI-BLAST) was employed to uncover distant relatives of the GSU1497 protein (Altschul, 1999).

To analyze the characteristics of the truncated pilins in *Geobacter*, all the truncated and full-length pilins in *Geobacter* (species options) were collected by BLAST (blastp) searches against the nr database where GSU1496 pilin and Gura_2677 pilin (full-length pilin of *G.uraniireducens* Rf4) were used as query sequences. The Gura_2677 pilin sequence was adopted since its capability of extracellular electron transfer has already been verified by biological experiments (Tan et al., 2016).

We used PSI-BLAST to find the homologous protein of GSU1497, which is encoded in the same operon as pilA (iterative threshold = 0.001, gap open\extend costs = 11/1). We defined the homologous genes of the GSU1496 and GSU1497 as GSU1496-like and GSU1497-like genes respectively. The proteins encoded by the homologous genes were therefore defined as GSU1496-like pilins and GSU1497-like proteins. GSU1496-like pilins are truncated type IVa pilins (TFaPs). The word “like” means these proteins have similar sequence and structure composition.

Whole genome information could be applied to find pilins and to reveal the location relationships between GSU1946-like and corresponding GSU1497-like genes. Pilins acquired from these genomes with complete genome information in *Desulfuromonadales* were utilized to conduct multiple sequence alignment and structure analysis. However, to investigate the evolution relationships between truncated and full-length pilins, all possible pilin sequences in the nr database were found by BLAST searches with Gura_2677 pilin as the query sequence. The 100 sequences with the highest similarity to Gura_2677 were downloaded from the BLAST results, redundant sequences were deleted, and sequences with the trans-membrane domain that is a characteristic of TFPs were chosen (Gorgel et al., 2015). All the signal peptide sequences were removed to obtain the final dataset of pilin protein sequences. The corresponding 16S ribosomal RNA gene sequences of these strains were also downloaded from NCBI's 16S ribosomal RNA sequences (bacteria and archaea) database.

### Predicting and clustering type IVA pilins

The PilFind algorithm is an invaluable tool that has long been used to identify TFPs in bacteria (Imam et al., 2011). Although trained on pilins from Gram-positive bacteria, PilFind can also identify TFPs in Gram-negative bacterium with low false positive rates (Imam et al., 2011). For Gram-positive bacteria, PilFind identified 155 of the 160 curated putative type IV pilins within operons containing TFP biosynthesis genes. For Gram-negative bacteria, PilFind was able to identify 27 of 29 experimentally verified type IV pilins, highlighting the predictive potential of this software (Imam et al., 2011). A pilin-like protein is identified when the returned value of the PilFind algorithm is true.

Clustering TFPs involves grouping pilins in such a way that pilins in the same group (called a cluster) are more similar to each other than they are to pilins in other clusters (Trehard et al., 2016). Cluster algorithm was used to detect a Type IVa cluster from among the pilins predicted by PilFind (Rollefson et al., 2011). To cluster TFaPs, the process was divided into two steps: align sequences using MAFFT (mafft-7.221-win64 with algorithm L-INS-i) (Katoh and Standley, 2013), and built phylogenic tree with MEGA 6.06 (using the neighbor-joining method and bootstrap = 1000; Tamura et al., 2013).

### Genetic, structural, and phylogenetic analysis

WebLogo was developed by Gavin and Schneider to generate sequence logos that are graphical representations of the patterns within a multiple sequence alignment, and to assist in discovering and analyzing those patterns (Schneider and Stephens, 1990; Crooks et al., 2004). We implemented WebLogo to find conserved sites/area and the distribution characteristics of aromatic and charged amino acids in the predicted pilins. The predicted sequences of the GSU1496-like pilins and GSU1497-like proteins were aligned using the BioEdit software (Hall, 1999).

The QUARK, I-TASSER (Iterative Threading Assembly Refinement), and PHYRE2 algorithms have been developed for protein folding and protein structure prediction (Xu and Zhang, 2012; Kelley et al., 2015; Yang et al., 2015a). The structures of the GSU1496-like pilins, GSU1497-like proteins, and full-length pilins were predicted using QUARK (for pilins shorter than 200 amino acids) or I-TASSER (for pilins longer than 200 amino acids). PyMOL is a molecular graphics system for the visualization of 3D chemical structures (Hart et al., 2015).

Unrooted tree topology based on multiple alignments of the pilin amino acid or 16S ribosomal RNA gene sequences was obtained using the Maximum Likehood method in MEGA 6.06. Consistency of branching was tested using a bootstrap analysis with 1000 resamplings of the data using MEGA 6.06 (Tamura et al., 2013).

### Operon analysis

DOOR (database for prokaryotic operons) contains computationally predicted operons of all the sequenced prokaryotic genomes and is a useful bacterial resource (Mao et al., 2009). To determine whether GSU1496-like and corresponding GSU1497-like genes share one operon, the operons of these genes were searched with locus tag in this database.

## Results and discussion

### Prevalence of truncated pilins in sequenced genomes of *Desulfuromonadales*

The 11 sequenced *Desulfuromonadales* genomes were obtained from the RefSeq database (release 70) (Supplementary Table S1). PilFind detected 183 candidates TFPs in the 11 genomes, including TFPs, Type II Secretion System (T2SS) major pilins, and T2SS minor pilins (Supplementary Figure S1). A phylogenic tree was built (neighbor-joining, bootstrap = 1000) for the candidates TFPs, and only proteins annotated as TFaP or proteins that clustered with the annotated TFaPs were considered as TFaPs (Supplementary Figure S1) for the subsequent analysis. The TFaP cluster contained 11 pilins, six were annotated as pilin-related and the others as hypothetical (Table 1). There was only one type IV pilin in each genome. However, in other class, for example *P. aeruginosa*, there was a pilin island that includes a major pilin gene cluster. GSU1496 and seven truncated pilins (length < 100 amino acids, Table 1) were included in the TFaP cluster (Supplementary Figure S1). Hence, truncated pilins were prevalent (8/11) in the known genomes of *Desulfuromonadales* (Table 1).

**Table 1:** **The annotation associated the 11 predicted type IVa major pilins in ***Desulfuromonadales*****.

**Strain**	**Gene name**	**Length**	**Annotation in NCBI**
*G.sulmrreducens*.PCA (PCA)	GSU1496	90	Hypothetical protein
*G.sulfiirreducens*.KN400 (KN400)	KN400_1523	90	Hypothetical protein
*G.lovleyi* SZ (SZ)	Glov_2096	76	Pilin domain-containing protein
*G.metallireducens* GS-15 (GS-15)	Gmet_1399	69	Hypothetical protein
*G.bcmidjicnsis* Bern (Bern)	Gbem_2590	76	Geopilin
*G*.sp.M21 (M21)	GM21_1636	74	Pilin
*G*.sp.M18(M18)	GM18J492	74	Pilin domain-containing protein
*G.daltonii* FRC-32 (FRC-32)	Geob_3369	218	Hypothetical protein
*G.uraniireducens* Rf4 (Rf4)	Gura_2677	203	Hypothetical protein
*P.carbinolicus* DSM 2380 (DSM 2380)	Pcar_2144	196	Geopilin
*P. propionicus* DSM 2379 (DSM 2379)	Ppro_1656	74	Pilin domain-containing protein

*Pilins with < 100 amino acids (the length of the alpha-helix) were defined as truncated pilins. The short names of strains are depicted in parentheses*.

In *Geobacter*, the truncated pilins of *G. sulfurreducens* and *G. metallireducens*, which are capable of coupling the complete oxidation of organic compounds to the reduction of iron and other metals, have been verified experimentally (Lovley et al., 1993; Caccavo et al., 1994; Lovely, 2012; Lovley, 2012). Additionally, it has been suggested that the pili of *G. sulfurreducens* KN400, which are assembled by truncated pilins, might serve as biological nanowires that can transfer electrons from the cell surface to the surface of Fe(III) oxides (Reguera et al., 2005). The implication is that truncated pilins may be related to the capacity of extracellular electron transfer in these bacteria. However, the relationship between truncated and full-length pilins has not been reported previously.

### General characteristics of the truncated pilins in *Geobacter*

**Figure 1:**
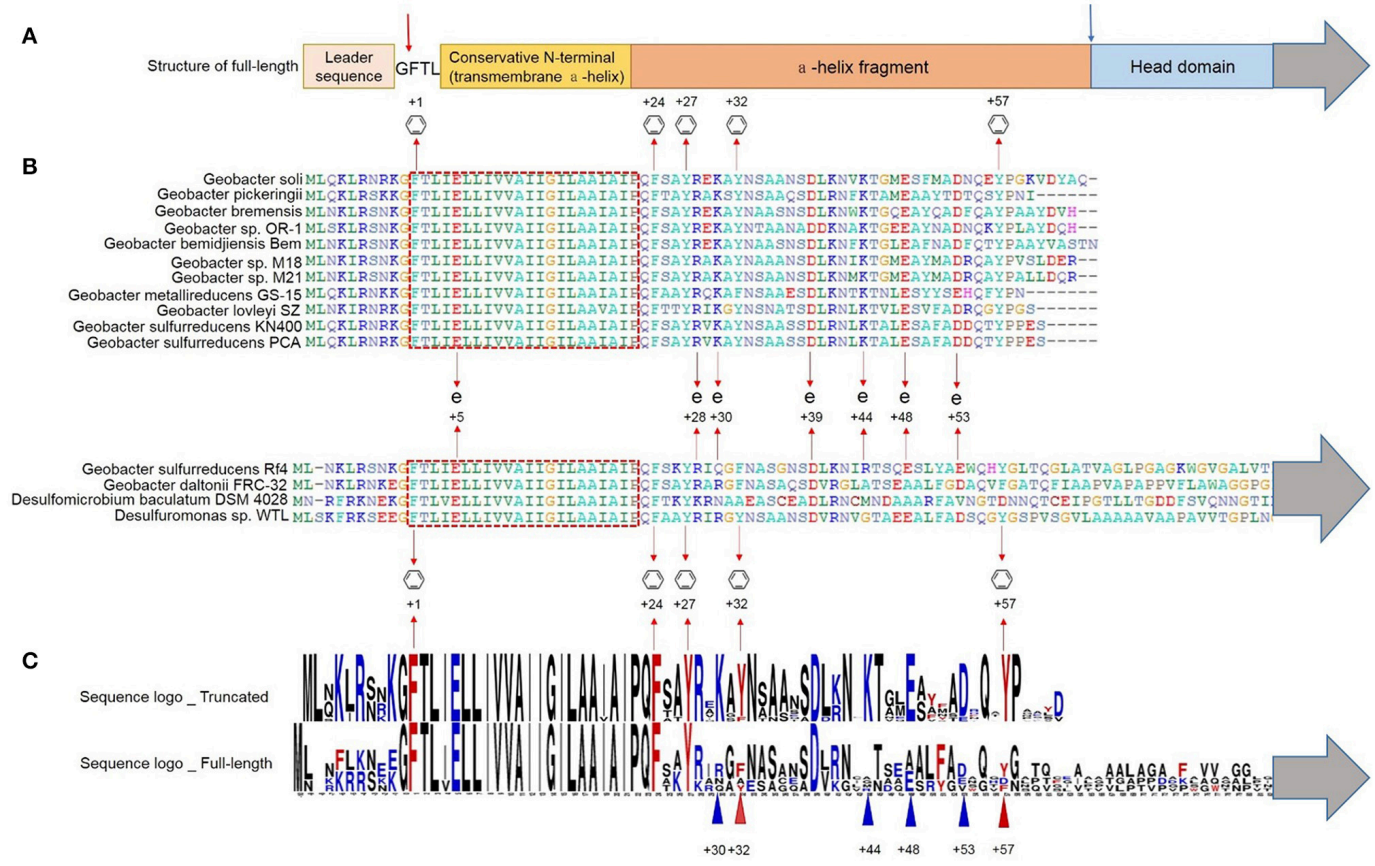
**Multiple alignment of truncated and full-length pilins of ***Geobacter*****. The gray arrows indicate the presence of the C-termini sequences of the full-length pilins (Supplementary Figure S2). **(A)** Schematic representation of the structure of a full-length pilin. The red arrow indicates the peptidase splice site, and the blue arrow indicates the truncation site. **(B)** The conserved transmembrane domain is shown in the red dotted box. 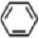 and + indicate the conserved aromatic amino acids and charged amino acids respectively. **(C)** Sequence logos of truncated (GSU1496-like) and full-length pilins in *Geobacter*. Red and blue triangles indicate aromatic amino acids and charged amino acids respectively.

To find all *Geobacter* pilins, including those in sequenced, and un-sequenced genomes, GSU1496 pilin and Gura_2677 pilin were used as query sequences in BLAST searches against the nr database to identify truncated and full-length pilins respectively. Four pilins (from *G. soli, G. pickeringii, Geobacter sp*. OR-1, and *G. bremensis*), which are not listed in Table 1, were found in *Geobacter* species (Figure 1A) with GSU1496 as the query sequence. Besides the pilins found using PilFind, no additional full-length pilins were found with Gura_2677 as the query sequence. Therefore, 11 truncated and two full-length pilins were identified in *Geobacter* species. For the comparison of full-length pilins in *Geobacter*, we included two additional pilins (from *Desulfomicrobium baculatum* DSM 4028 and *Desulfuromonas sp*. WTL) that are close homologs of Gura_2677. All the selected protein sequences were aligned using BioEdit and sequences logos were built with WebLogo (Hall, 1999).

The multiple alignment shows that all the pilins (full-length and truncated) had the conserved N-terminal signal sequence with a highly conserved hydrophobic helix (Figures 1A,B), which is consistent with them both being TFPs (Gorgel et al., 2015). The distinctive transmembrane domain (the red dotted box in Figure 1B) is also a hallmark of TFPs, which also acts as a conserved structure domain in protein interactions (Giltner et al., 2012). The main role of this transmembrane domain is to fix the membrane protein into the cell membrane. Moreover, pilins have a conserved peptidase splice site (red arrow in Figure 1A) upstream of the transmembrane domain. To form a mature pilin, the signal sequence is cleaved off at this peptidase splice site (Strom and Lory, 1991).

Several conserved aromatic amino acids (at positions +1, +24, +27, +32, +57) were detected in the truncated protein sequences (Figure 1B). Highly conserved sequences are generally considered to have specific functions, including stability, reproduction, and microbial functions (Robson et al., 1988). Aromatic amino acids [phenylalanine (F), tyrosine(Y)] are known to be required for pili conductivity and long-range extracellular electron transfer in *G. sulfurreducens* (Vargas et al., 2013). TFPs assemble to form symmetrical helical structures (Burrows, 2014). In a polymeric pilus structure, truncated monomers may assemble tightly together so that the conserved aromatic amino acids from different monomers are closely packed, resulting in pi-pi interactions that contribute to pili conductivity. There are also many non-conserved aromatic amino acids between positions +50 and +57, including F51 of *G. sulfurreducens* PCA/KN400, Y50, Y51, and F56 of *G. metallireducens* GS-15, and F43, F50, F54, and Y61 of *G.bemidjiensis* Bem. The conserved and non-conserved aromatic amino acids are mainly distributed in two areas: +24 to +32 and +50 to +61 (Figure 1). The conserved aromatic amino acids may induce the conductivity of pili, while the non-conserved aromatic amino acids may introduce small distances between the aromatic rings, which may contribute to the extracellular electron transfer capacity of some pili.

In the truncated pilins of *Geobacter*, the conserved amino acids include not only the aromatic amino acids, but also charged amino acids [glutamic acid (E), aspartic acid (D), lysine (K), and arginine (R)] (Figures 1B,C). The conserved charged amino acids (E5, R28, K30, D39, K44, E48, and D53) possibly form salt bridges when the monomers assemble to form polymers. Although the electronic interactions in salt bridges are relatively weak, small stabilizing interactions can add up and make an important contribution to the overall stability of a conformer. Furthermore, the conserved charged amino acids are distributed in the same regions as the aromatic amino acids (+24 to +32 and +50 to +61). Salt bridges in this region might affect the configuration of the aromatic residues and their reduction potential, thus influencing the rates of electron transfer.

By comparing the truncated and full-length pilin sequences, we found that phenylalanine (F) occupied the +32 site in almost all the full-length pilins, while tyrosine (Y) occupied the +32 site in the truncated pilins; further, Y57, K30, K44, E48, and D53 were not conserved in the full-length pilins, but conserved in the truncated pilins (Figure 1C). Phenylalanine and tyrosine differ only in one OH group, which could be easily modified by glycerophosphate that plays a significant role in the process of adsorption and combination with ferric iron ions (Reguera et al., 2005). Moreover, the reorganization energy that contributed to the electron transfer activation energy of tyrosine was reported to be stronger than it was for phenylalanine (Yan et al., 2015). When pi-pi interactions are forming, the more electron transfer activation energy there is, the better the conductive effect the aromatic amino acids may have (Zhuang and Wang, 2015). Feliciano et al. (2015) reported that two mutations (Y32A (alanine), and Y57A) in the pilin amino acid sequence severely affected the function of pili as an electronic conduit *in vivo*. Therefore, the two amino acids at positions +32, and +57 probably generate pi-pi interactions with other aromatic amino acids, and then contribute to long-range extracellular electron transfer with the conserved charged amino acids in truncated pilins. In the previous study (Feliciano et al., 2015), the type, configuration, and distances of the aromatic amino acids in aromatic contacts and the pilus surface properties were different in an aspartic acid (D) 53A mutant. Hence, it can be inferred that the conserved charged amino acids, K30, K44, E48, and D53, in truncated pilins of *Geobacter* may play important roles in monomer assembly and the formation of pi-pi interactions.

The truncated pilins in *Geobacter* have similar amino acid sequence lengths and the truncation sites (blue arrow in Figure 1A) are all in the loop region that is downstream of the alpha-helix. It can be inferred that the fission site in full-length pilins is located in this loop area, assuming truncated pilin evolved from full-length pilin. The sequences downstream of the alpha-helices in full-length pilins are not conserved (Figure 1, Supplementary Figure S2), which may explain why only truncated pilins have been found to be conductive, up to now.

### Structural features of GSU1496-like pilins and GSU1497-like proteins in *Geobacter*

**Figure 2:**
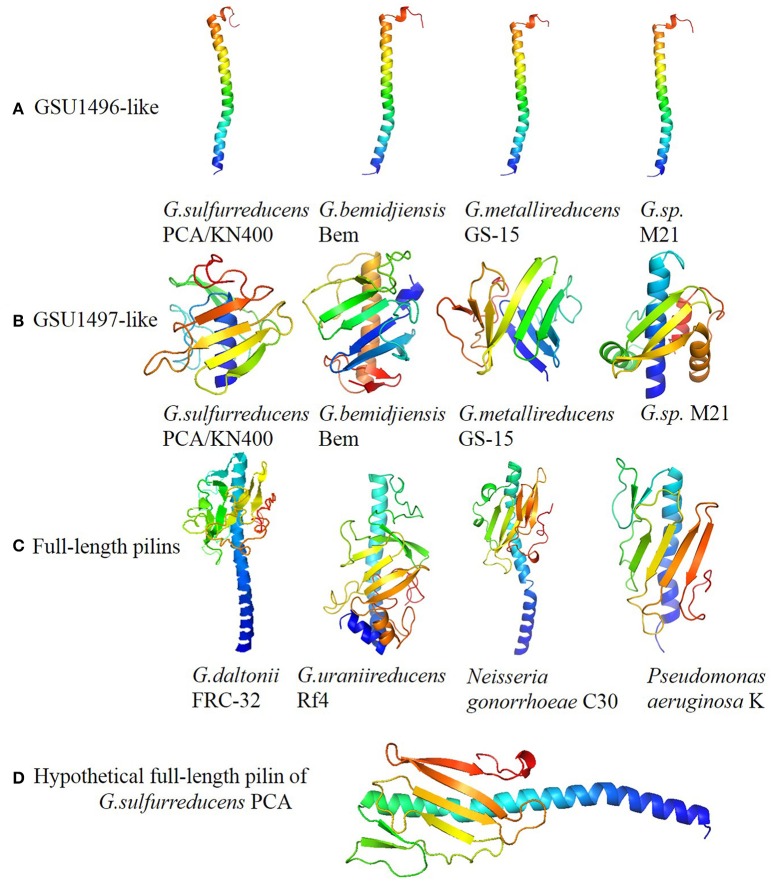
**Panels (A–D)** represent structures of GSU196-like proteins, GSU1497-like proteins, full-length pilins and hypothetical full-length pilin of *G. sulfurreducens* PCA, respectively. The structures were predicted by QU ARK and I-TASSER, except for GSU1496 (PDB: 2M7G). Five GSU1496-like pilins and five GSU1497-like proteins are shown in Figure 2. The others are shown in Supplementary Figure S3.

The eight GSU1496-like pilins listed in Table 1 were obtained by combing PilFind with cluster analysis. The eight GSU1497-like proteins were found by PSI-BLAST with the GSU1497 amino acid sequence as the query, because the GSU1497 gene is located directly downstream of GSU1496 gene and in the same operon (Methe et al., 2003; Holmes et al., 2006). The tertiary structures of GSU1496-like pilins and GSU1497-like proteins in *Geobacter* were predicted by QUARK (<200 amino acids) and I-TASSER (>200 amino acids). We found that all the GSU1496-like pilins contained an alpha-helix, while all GSU1497-like proteins contained a group of consecutive anti-parallel beta-sheets. The full-length pilins all had a complete alpha-helix and a C-terminal globular domain, regardless of whether the structure was obtained from biological experiments or *ab initio* calculations (Figure 2, Supplementary Figure S3). For example, the full-length pilins of *G. daltonii* FRC-32 and *G. uraniireducens* Rf4 both have the N-terminal alpha-helix and the C-terminal globular domain (Figure 2). The consecutive anti-parallel beta-sheets that were characteristic of the GSU1497-like proteins were also part of the C-terminal globular domain of these two full-length pilins.

The spatial structures of the GSU1496-like pilins, GSU1497-like proteins, and full-length pilins from *Geobacter* are shown in Figure 2 and Supplementary Figure S3. Obviously, the structural composition of the GSU1496-like pilins (Figure 2A) and GSU1497-like proteins (Figure 2B) are similar to the N-terminal alpha-helix and C-terminal consecutive anti-parallel beta-sheets regions of the full-length pilins (Figure 2C), respectively. Moreover, we obtained a hypothetical full-length pilin by joining the GSU1496 major pilin sequence (61 amino acids) and the GSU1497 protein sequence (111 amino acids with the signal peptide removed). Then, we utilized PHYRE2 (Kelley et al., 2015) to predict its structure (Figure 2D). The structural composition (alpha-helix and consecutive anti-parallel beta-sheets) and the length of the hypothetical protein are similar to those obtained from biological experiments of full-length pilins from *N. gonorrhoeae, P. aeruginosa* and *Vibrio cholera* (Figure 2C and Supplementary Figure S3). These results indicate that a GSU1496-like pilin and a corresponding GSU1497-like protein possibly originated from one full-length pilin gene.

It is known that the structure of a protein determines its biological function. The diversity of type IV pilin structures determines their functional diversity. Truncated and full-length pilins have distinct surface characteristics, resulting in interaction with different molecules. The assembly of pilin monomers into type IV pili could create a polymer machinery which mediates diverse cellular functions, including cell signaling, host-cell adhesion, DNA transfer, surface motility, microcolony, and biofilm formation, macromolecule degradation, electron transport, and pathogenesis (Craig et al., 2004). For example, *N. gonorrhoeae* pili can evokes the host immune response and is potential drug and vaccine targets. Additionally, the C-terminal global domain of full-length pilin is an important region for pilus interaction with other pili, host cell receptors and immune components (Forest et al., 1999). For instance, the surface motility of C-terminal of *N. gonorrhoeae* pilin (Figure 2C) can help bacteria across semi-solid surfaces such as the mucosal epithelia (Zaburdaev et al., 2014).

However, the truncated pilin have no global domain in C-terminal. This structure character could be result in special biochemical property of pilin. For example, the pilus assembled by truncated pilin of *G. sulfurreducens* KN400 has metallic-like conductivity (Malvankar et al., 2011). This metallic-like conductivity relies on the structures of the *G. sulfurreducens* pilus in which aromatic amino acids may promote long-distance electron transport (Malvankar et al., 2015). Pilus is a kind of polymer consisting of thousands copies of pilins. When a microorganism uses ATP-powered machinery to assemble a polymeric pilus structure, truncated pilins may be assembled tightly together so that aromatic amino acids from different monomers could construct pi-pi interactions that contribute to pili conductivity. This mechanism for long-rang electron transport is well known as a potential reason for pili conductivity.

### Truncated pilins probably evolved from full-length pilins

To study the origin of the truncated pilin, we analyzed the genetic relationship between truncated and full-length pilins. Gura_2677 was employed in BLAST searches to find all known truncated and full-length pilins because, with GSU1496 as the query sequence, only truncated pilins will be obtained and no pilins were obtained by using GSU1497. Additionally, in three full-length pilins (Table 1), Gura_2677 and Geob_3699 are belong to *Geobacter* which is a typical genus with conductive pilins. Pili assembled by Gura_2677 pilin are capable of extracellular electron transfer, which has already been verified by biological experiments. There are biased of final list of pilins, when we utilized Geob_3699 pilin sequence to search for homologs. However, the bias in the final list of pilins was acceptable, because two searched results shared identical set of sequence in the top 60 similar sequences and have few difference (<13%) in top 100 similar sequences. The 100 most similar protein sequences were downloaded for phylogenetic analysis. After deleting redundant sequences, 80 pilin sequences (41 truncated major pilins and 39 full-length pilins) were obtained. The 11 truncated and two full-length pilins sequences from *Geobacter* were included in the 80 pilin sequences for the analysis.

#### Species distribution of truncated pilins

The species of the 80 selected pilin sequences were classified at the order level (Supplementary Figure S4). Seventy eight of the pilins belonged to 23 families from six phyla; the remaining two pilins were atypical genera in prokaryotes and were not classified. Most pilins (66) were *Proteobacteria*, which is the main phylum that contains bacteria capable of extracellular electron transfer (Logan, 2009) Strains with truncated pilins were distribute in a limited number of families, namely *Deferribacter, Flexistipes, Streptococcus, Candidatus Accumulibacter, Neisseriaceae, Thermithiobacillus, Desulfobacterales, Desulfuromonadales, and Pseudomonas*. Strains with full-length pilins were distribute more widely, but were also present in the same families as truncated pilins. The species distribution of these pilins suggests that the truncated pilins may have an evolutionary relationships with full-length pilins.

**Figure 3:**
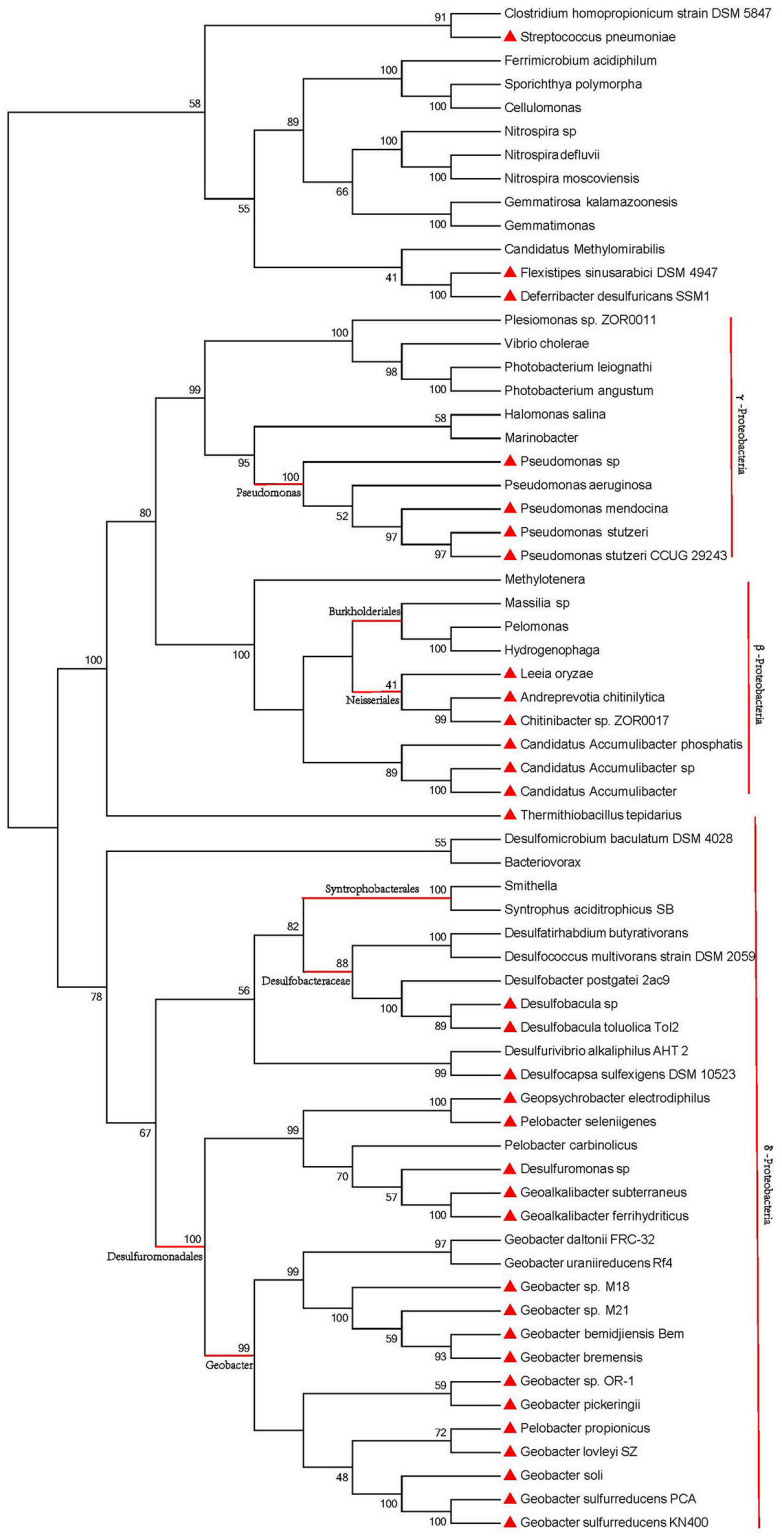
**Phylogenetic analysis of 16S ribosomal RNA sequences for species containing pili**. Red triangles represent strains with truncated pilins. Pilins from other unlabeled strains are full-length. Red vertical lines and horizontal lines mark the different *Proteobacteria* classes and different genus of *Proteobacteria*. The genus of *Proteobacteria* are highlighted by red horizontal lines.

16S ribosomal RNA gene sequence has been widely used for phylogenetic studies, as it is highly conserved between different species of prokaryotes (Naga et al., 2013). The corresponding 16S ribosomal RNA gene sequences of the 80 strains were utilized to build the phylogenetic tree (GTR+G+I, bootstrap = 1000, cut-off for condensed tree = 20%), as shown in Figure 3. The phylogenetic tree of species contains two major lineages: non-*Proteobacteria* and *Proteobacteria*. The *Proteobacteria* lineage consists of γ-*Proteobacteria*, β-*Proteobacteria*, and δ-*Proteobacteria* branches. The phylogenetic tree (Figure 3) showed that strains in one species are always clustered in one branch. Strains with a truncated pilin have a close genetic relationship, no matter in γ-*Proteobacteria*, β-*Proteobacteria*, or δ-*Proteobacteria* branches. Genetic relationships among the pilin-truncated strains from a same genus, e.g., *Geobacter*, were stronger than those between the pilin-truncated strains and the full-length-pilin strains. Moreover, in one *Proteobacteria* class branch, strains with full-length pilins showed closer genetic relationship to other *Proteobacteria* classes than those with truncated pilins. According to these results, we can infer that full-length pilin, rather than truncated pilin, may appear first in the evolutionary process of *Proteobacteria*.

#### Truncated pilins evolved from full-length pilins

**Figure 4:**
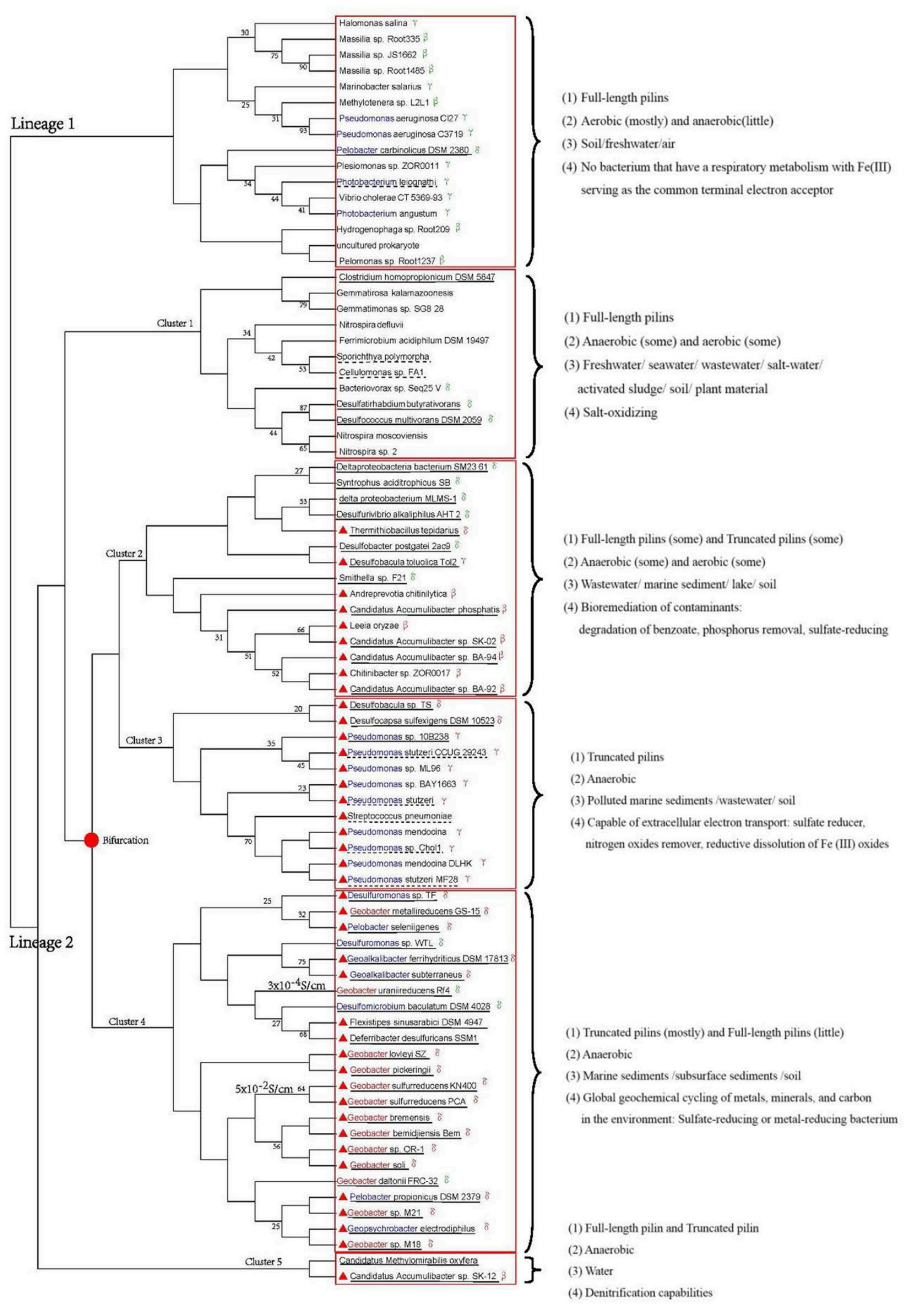
**Phylogenetic analysis of pilin amino acid sequences**. Red solid line boxes mark the different clusters. Blue letters indicate genera that including electricigens, except for *Geobacter* (red letters). Red triangles indicated strains with truncated pilins. The *Proteobacteria* class is shown after the corresponding species name. Bifurcation of truncated pilin evolution is marked with a solid red dot. Dashed and solid lines under the strain names indicate facultative anaerobic and anaerobic bacteria, respectively. Characteristics of each cluster, i.e., types of pilins, growth environments, habitats and functions, are provided on the right of the tree. The conductivity of *G. uraniireducens* Rf4 and *G. sulfurreducens* KN400 pili are on the left of strain names.

Phylogenetic analysis can help reveal valuable information as about the origin, evolution, and potential function of genes (Baldauf, 2003). We used sequences from 80 strains to explore the evolutionary origin of the truncated pilins. A consensus tree was built using MEGA 6.06 (LG+G, bootstrap = 1000, cut-off for condensed tree = 20%), as shown in Figure 4. Although the bootstrap values in deep branch could achieve 93, the bootstrap values in shallow branch were not very good. The phylogenetic tree for pilins (amino acids from 57 to 228) demonstrated very low bootstrap values, likely due to the relatively small size of the alignment, the high degree of sequence similarity in the highly conserved genome region (N-terminal sequences) and the diversity of C-terminal sequences of full-length pilins (Supplementary Figure S2). Therefore, the low bootstrap values were understandable, and the topology of phylogenetic tree was correct. We used information from the literatures and gene annotations to identify electricigens in the *Proteobacteria* (Supplementary Table S2).

In Figure 4, the strains from one species are distributed in multiple branches. However, in phylogenetic tree of species, they are clustered in one branch (Figure 3). This situation revealed the sequences of pilins might undergo variation through horizontal gene transfer between genera in *Proteobacteria*. The horizontal gene transfer likely occurred in cluster 2–4. Moreover, the distribution of pilins in the phylogenetic tree (Figure 4) represents the genetic relationship between truncated and full-length pilins. Two lineages are shown in Figure 4, one representing full-length pilins (Figure 4, lineage 1), and the other consisting of both full-length pilins and truncated pilins (Figure 4, lineage 2). These genetic relationships suggested that truncated pilins evolved from full-length pilins.

Strains with truncated pilin are distributed in four clusters, namely cluster 2, 3, 4, and 5 of lineage 2. Clusters 2, 4, and 5 also contain full-length pilins, which implies the closely evolutionary relationships between truncated and full-length pilins in these clusters, as well as similar functions. For example, *Desulfobacula toluolica* Tol2, *Desulfobacter postgatei* 2ac9, and *Thermithiobacillus tepidarius* in cluster 2, and *Candidatus Methylomirabilis oxyfera* and *Candidatus Accumulibacter sp*. SK-12 in cluster 5 are all known to play important roles in bioremediation of contaminants (Seviour et al., 2003; Wöhlbrand et al., 2013; Yang et al., 2015b). *G. uraniireducens* Rf4, *G. daltonii* FRC-32, *G. sulfurreducens* KN400, *Pelobacter propionicus* DSM 2379, and *Geopsychrobacter electrodiphilus* of cluster 4 are all in order *Desulfuromonadales*. These relationships indicate that the evolution of truncated pilins from full-length pilins may have been a gradual process. According to the structural analysis (Figure 2) and phylogenetic trees (Figures 3, 4), it can be inferred that the pilin evolutionary process consisted of three stages. We hypothesize that all the TFPs were full-length pilins at the beginning, as indicated by lineage 1 and cluster 1 of lineage 2. Then, some truncated pilins appeared in a piecemeal way, as indicated by cluster 2 of lineage 2. Finally, under evolutionary pressure, the truncated pilins grouped into specific genera, as indicated by cluster 3 and cluster 4 of lineage 2. The deduced bifurcation of truncated pilin evolution is indicated by the solid red dot in Figure 4.

The phylogeny of pilin sequences did not match the phylogeny of 16S ribosomal RNA sequences (Figures 3, 4). The phylogeny of 16S ribosomal RNA sequences indicated the evolutionary relationship of species. The phylogeny of pilin sequences probably suggested the relationship of functions for pilins. Pilins from different genera could have similar function, because the horizontal gene transfer happened. Pilins distributed in one branch probably have common ancestor or similar functions (Pontarotti, 2009). Therefore, the pilin clades could be grouped by characteristics, e.g., types of pilins, growth environments, habitats, and functions. To study the similarities of strains with truncated pilins, the functions, and biochemical properties common to the strains in each branch were examined by reading literatures and gene annotations (Figure 4, Supplementary Tables S3, S4). These informations suggested that most strains with truncated pilin in Figure 4 are sharing a common function, i.e., capability of transferring electrons to extracellular acceptors.

Among the bacteria in clusters 2, 3, 4, and 5 there are some strains with full-length pilin; however, the exoelectrogenic activities of these strains are possibly lower than the strains with truncated pilins, according to previous studies that indicated only the truncated pilin of *G. sulfurreducens* PCA was known as a metal-like conductive pilin (Lovley and Malvankar, 2015). The mechanism of this exoelectrogenic activity may be explained as follows: the smaller C-terminal domains of the truncated pilins may make it easier for them to form pi-pi interactions because they could potentially form more tightly packed assembly modes than the full-length pilins. Therefore, the truncated pilins are probably more prominent than full-length pilins in extracellular electron transporting. Pilus assembled by truncated pilin perhaps is an effective way for microorganisms to transfer electrons to extracellular acceptors in extreme environments. For example, as shown in Figure 4, the conductivity of the *G. uraniireducens* Rf4 pili (consists of full-length pilins, 3 × 10^−4^S/cm) was much lower that the conductivity of the *G. sulfurreducens* KN400 pili (consists of truncated pilins, 5 × 10^−2^S/cm) (Tan et al., 2016).

Many anaerobic bacteria (include a lot of sulfate-reducing and metal-reducing bacteria) with truncated pilins (clusters 2, 3, 4, and 5 in Figure 4) are capable of long-range extracellular electron transfer (Supplementary Tables S3, S4). These anaerobic microbes have two characteristics in common: they were isolated in extreme environments, such as sea floor sediments and wastewater, and they play important roles in global geochemical cycling of metals, minerals, and carbon in the environment, bioremediation of contaminants, and microbe-electrode interactions. Because oxygen and other electron acceptors are depleted during organic matter degradation in these extreme environments, it can be inferred that truncated pilins play important roles in the long-range extracellular electron transfer process. These extreme environments could promote electron transfer of truncated pilins to extracellular acceptors.

For most strains with truncated pilins (include many sulfate-reducing and metal-reducing bacteria) in clusters 2, 3, 4, and 5 (Figure 4), they have been reported that shuttles or redox-active proteins play limited roles in transferring electrons to extracellular acceptors. For instance, c-type cytochromes do not appear to play an important role in long-range electron transport along pili of *G. sulfurreducens* (Leang et al., 2010; Lovely, 2012; Malvankar et al., 2012). Microorganisms that do not have direct contact with external electron acceptors, possibly complete the long-range electron transfer through pili with metallic-like conductivity, such as *G. sulfurreducens* KN400, which has a truncated pilin (Lovley, 2012). In these bacteria, truncated pilins possibly evolved to carry out long-range electron transport.

Therefore, the evolution of pilins is probably a result of the pressure of extracellular electron transfer for anaerobic bacteria that do not have direct contact with external electron acceptors. Our phylogenetic analyses allow us to infer that truncated pilins evolved from the full-length pilins. A limitation of the phylogenetic tree is the small amount of sequencing data that was available. Despite this, the tree described here indicates that truncated pilins probably evolved from full-length pilins in bacteria that are capable of extracellular electron transfer.

### GSU1496-like pilins appear along with GSU1497-like proteins, and the possible fission process of the full-length pilin gene

From the structural and phylogenetic analyses, we inferred that GSU1496-like and GSU1497-like genes probably evolved from the N-terminal and C-terminal encoding regions of a full-length pilin gene, respectively. To analyze the process of gene fission, we studied the location relationship between GSU1496-like and GSU1497-like genes. Whole genome information is available for only eight *Geobacter* strains (Table 1). Eight GSU1496-like and eight GSU1497-like genes were extracted from the eight genomes using PilFind and PSI-BLAST.

#### GSU1496-like and GSU1497-like are adjacent to each other in the geobacter genome

**Figure 5:**
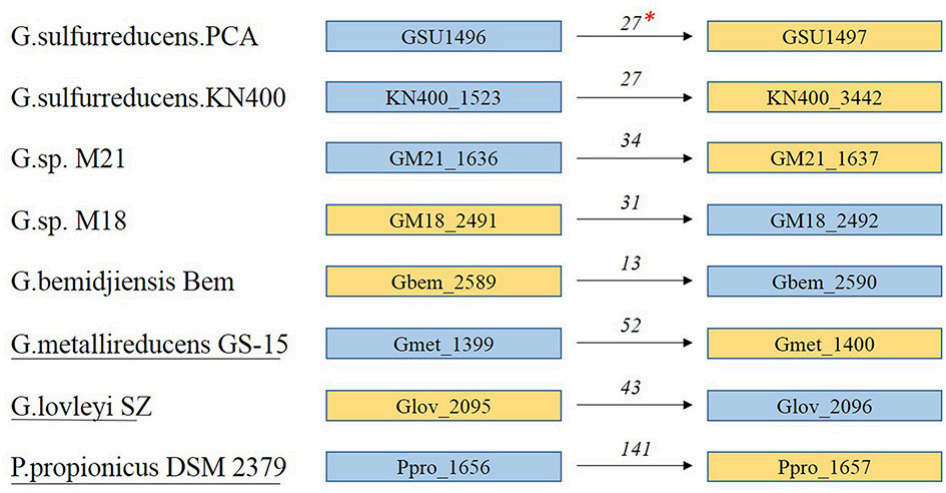
**Locus tags and location of GSU1496-like and GSU1497-like genes in eight genomes**. ^*^The numbers above the arrows indicates the distance (base pairs) between the genes encoding the GSU1496-like pilin and the GSU1497-like protein. Blue boxes indicate locus tags of GSU1496-like genes, and yellow boxes indicate GSU1497-like genes. The arrows indicate the order of the genes in the genome. The strain names without or with underlines indicate the two genes do or do not share one operon respectively.

Locus tags (e.g., GSU1496 and GSU1497) generally represent the positions of genes in a genome (Supplementary Tables S5, S6). In the genomes shown in Figure 5, GSU1496-like and GSU1497-like genes are adjacent to each other (Supplementary Table S6). Therefore, the genes encoding GSU1496-like pilins and GSU1497-like proteins appear together in all these genomes.

GSU1497-like proteins and the corresponding GSU1496-like pilins may share a relationship because they are adjacent in the genome and, in most cases, share the same operon (Richter et al., 2012). In *G.sulfurreducens* PCA, the length (90 + 124 amino acids) of the two proteins (GSU1496 and GSU1497) is similar to the lengths of the full-length pilins in *Geobacter* (e.g., *G. daltonii* FRC-32 and *G. uraniireducens* Rf4; Table 1). Similar correlations also exist in other strains of *Geobacter* with truncated pilins (Table 1, Supplementary Tables S5, S6). This property again supports our hypothesis that the gene encoding full-length pilins in *Geobacter* probably split into two parts, pilA-C (GSU1496-like) and pilA-N (GSU1497-like).

#### Fission mechanism of full-length pilin gene

**Figure 6:**
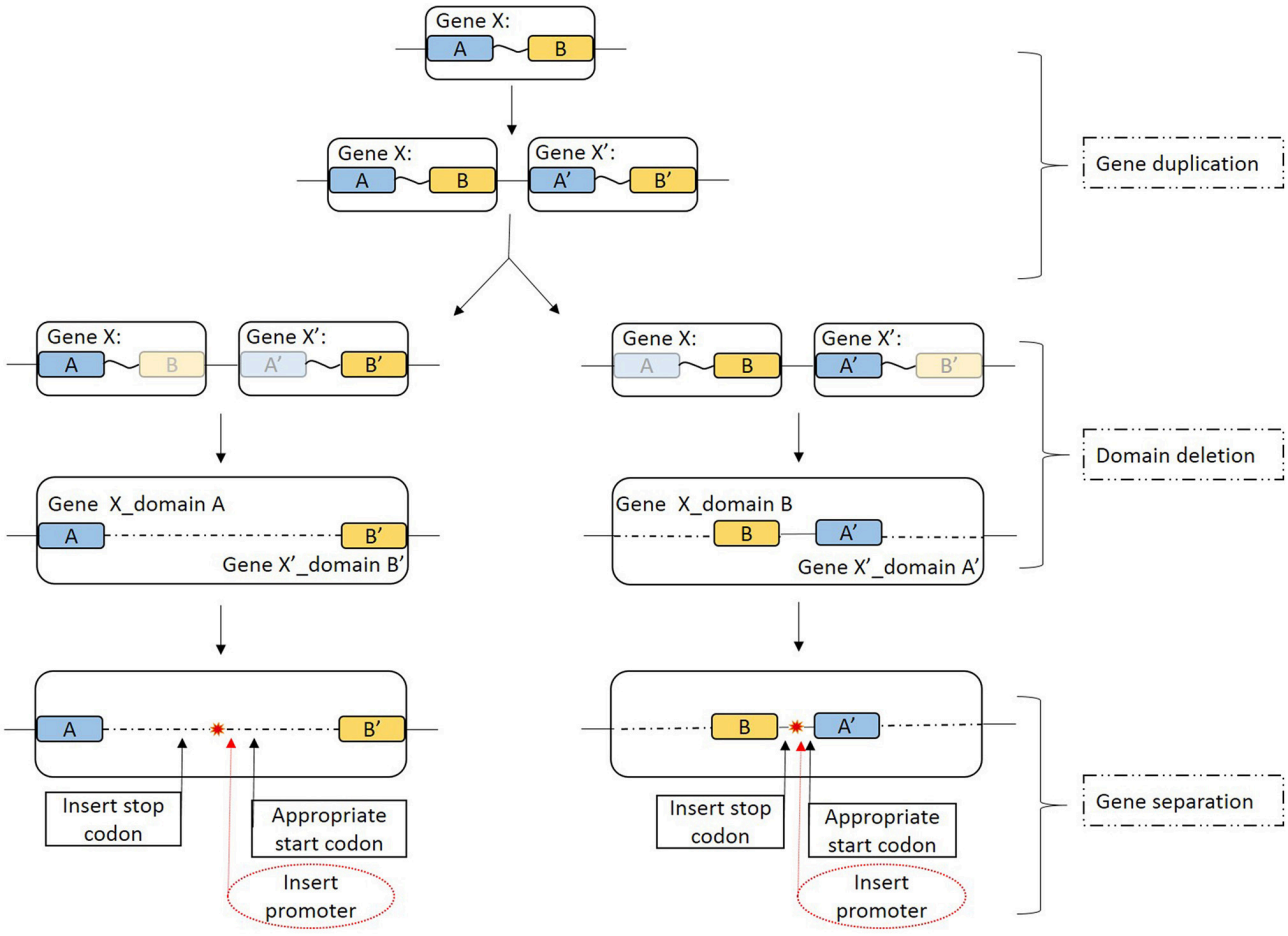
**Schematic view of the possible full-length pilin gene fission mechanism**. A description of each stages is given on the right-hand side. Gene X is a full-length pilin gene; A and A′ represent the GSU1496-like domain; and B and B′ represent the GSU1497-like domain. Blue and yellow boxes indicate the GSU1496-like and GSU1497-like domains, respectively. Light blue and light yellow boxes indicate corresponding degenerative domains. The large boxes that include the blue and yellow boxes indicate the area of full-length pilin gene in the genome. The arrows indicate possible details of the gene fission processes. Red dashed ovals indicate the insert promoter step, which is not necessary in this case because in an operon the genes are controlled by one promoter. The red star indicates that the full-length pilin gene has been split into two separate genes.

Because the GSU1497 and GSU1496 genes were found to be in the same operon (Richter et al., 2012), we used DOOR to determine whether the same was true for GSU1496-like and GSU1497-like genes in other genomes. Five strains were found where it was true, and three strains were found where it was not true (underline in Figure 6). However, the order of the two genes is reverse in *Geobacter sp*. M18, *G.bemidjiensis* Bem and *G. lovleyi* SZ.

The diversity of TFPs is because of shufflon rearrangements or other recombination events within species and strains (Giltner et al., 2012). Recombination events such as translocation, inversion, or segmental duplication can cause the accidental fission of a gene into several parts. Previous studies have been strongly contradict the idea that fissions occur at low relative rate, because an alternative mechanism was proposed that may drive gene fission (Baldauf, 2003; Durrens et al., 2008; Leonard and Richards, 2012). Gene fission is now considered to play an important and hitherto underestimated role in gene evolution. Leonard et.al (Leonard and Richards, 2012) identified three theoretical mechanisms that, individually or collectively, could result in gene fission: (Malvankar and Lovley, 2012) fission by separation (a stop codon, a promoter region, or a start codon inserted into the open reading frame at selectively viable positions, resulting in two separate genes); (2) fission by degeneration (loss of function and degeneration of the sequence encoding one domain); and (3) fission by duplication (duplication of a gene fusion and differential loss of constituent domains by either the first or the second mechanism). These three mechanisms could lead to gene fission either separately or in combination.

First, the full-length pilin gene (Gene X) is duplicated (Figure 6, gene duplication). For instance, in *P. aeruginosa*, there is a pilin island that includes a major pilin gene cluster (Giltner et al., 2011). Furthermore, the structure of the noncore minor pilin PilX from *Neisseria meningitides* is similar to that of its major pilin (Helaine et al., 2007). Then duplicated genes (Gene X and Gene X′) can degenerate if the sequence encoding one similar domain (GSU1496-like or GSU1497-like) is deleted because functional selection ceased (Leonard and Richards, 2012). Degeneration might result in two domains that are inversed (Figure 6, domain deletion), which might explain why although the GSU1496-like and GSU1497-like genes are adjacent, the directions are sometimes reversed. Finally, GSU1496-like and GSU1497-like genes are produced due to gene fission by separation (Figure 6, gene separation). In the separation stage, the insertion of a promoter is not necessary because an operon is a functioning unit of genomic DNA that contains a cluster of genes under the control of a single promoter (Wells et al., 2016). These processes result in different length gaps between the GSU1496-like and GSU1497-like genes. The results of the phylogenetic, genetic, and structural analyses, together indicate that truncated pilins probably arose from the full-length pilins.

## Conclusions

Analysis of complete genome sequence data deposited in RefSeq 70 revealed that there are many truncated and few full-length pilins in *Desulfuromonadales* species. In this study, we have presented a bioinformatics description for truncated pilins and GSU1497-like proteins. Genetic, structural, and phylogenetic properties were calculated, and gene relationships, conservative structures and sequences, and the evolution of truncated pilins were revealed.

Truncated pilins were found to have a distinctive N-terminal signal sequence and a highly conservative hydrophobic spiral that contained not only a transmembrane domain, but also a conservative structural domain that is involved in protein interactions, as reported previously in TFPs (Giltner et al., 2012). Furthermore, GSU1496-like pilin sequences have a conservative peptidase splice site, two aromatic clusters (+24 to +32 and +50 to +61), several conservative aromatic amino acids (F1, F24, Y27, Y32, and Y57), and charged amino acids (E5, R28, K30, D39, K44, E48, and D53). Among these amino acids, K30, Y32, K44, E48, D53, and Y57 are distinctive conserved residues in truncated pilin sequences.

The aromatic amino acids in the truncated pilins may help them easily form pi-pi interactions than full-length pilins, because of the symmetric assembly mode of TFPs. The smaller size of truncated pilins might allow the polymers to be more tightly packed. Charged amino acids could form salt bridges that might affect the configuration of the aromatic residues and their reduction potential, which, in turn, would influence the rates of electron transfer. Therefore, the distinctive conserved amino acids in truncated pilins may have an influence on the conductivity of pili, as was shown in a study using five mutated aromatic amino acid (Vargas et al., 2013). Short sequence lengths may be a distinctive feature of metal-like conductivity pilins.

The structures of GSU1496-like pilins contain a segment of alpha-helix, while the main conservative feature of GSU1497-like proteins is consecutive anti-parallel beta-sheets. The full-length pilins of *G. daltonii* FRC-32 and *G. uraniireducens* Rf4 have both an N-terminal alpha-helix segment and a C-terminal globular domain. The structural composition and sequence length of a hypothetical full-length pilin of *G. sulfurreducens* PCA/KN400 are similar to those of the native full-length pilin obtained from biological experiments. This finding showed that the structures of GSU1496-like pilins and GSU1497-like proteins are similar to the N-terminal and C-terminal structures of full-length pilins, respectively. We concluded that GSU1496-like pilins and GSU1497-like proteins in *Geobacter* probably arose from the full-length pilins.

The genetic features, structural characteristics, and unrooted bootstrap tree determined in this study, allowed us to infer that strains with truncated pilins are capable of extracellular electron transfer and that the truncated pilins might have evolved from full-length pilins. Furthermore, GSU1496-like and GSU1497-like genes are adjacent in the genome, although sometimes the directions are reversed and sometimes they share the same operon. These properties indicate that there is a close relationship between the two genes. Our findings correspond with those of Richter (Richter, 2011) who reported that GSU1497 was absolutely essential to stabilize PilA in *G. sulfurreducens* PCA. We can infer that the fission of a full-length pilin gene may have resulted from gene duplication and degeneration, giving rise to the GSU1496-like and GSU1497-like genes in *Geobacter*. These results show that the conductive pilins have specific sequence features, structural characteristics, and evolutionary origins, and provide new insights into the mechanism of pili conductivity.

## Author contributions

CS designed the study, carried out the study and drafted the manuscript; KX and QY helped write the manuscript; XS conceived of the study and was the lead writer of the manuscript. All authors read and approved the final manuscript.

## Funding

The research was sponsored by the National Natural Science Foundation of China (No. 61472078) and the Open Research Fund of State Key Laboratory of Bioelectronics, Southeast University, China.

### Conflict of interest statement

The authors declare that the research was conducted in the absence of any commercial or financial relationships that could be construed as a potential conflict of interest.
