# Corrigendum: Comparative Analysis of Type IV Pilin in *Desulfuromonadales*

**DOI:** 10.3389/fmicb.2017.00342

**Published:** 2017-03-02

**Authors:** Chuanjun Shu, Ke Xiao, Qin Yan, Xiao Sun

**Affiliations:** State Key Laboratory of Bioelectronics, School of Biological Science and Medical Engineering, Southeast UniversityNanjing, China

**Keywords:** conductive pilin, extracellular electron transfer, structure, phylogenetic analysis, gene fission

In the original article, we did not notice the newest publication of Lovley group (Holmes et al. paper) when we submitted our manuscript, which previously demonstrated that the electrically conductive pili of *Geobacter* are recently evolved (Holmes et al., 2016). Here, the authors acknowledge the previous contributions of Lovley group and apologize for this oversight.

This error does not change the scientific conclusions of the article in any way.

## Conflict of interest statement

The authors declare that the research was conducted in the absence of any commercial or financial relationships that could be construed as a potential conflict of interest.
