# Supplementary material for: Comparative Analysis of Type IV Pilin in Desulfuromonadales
Source: Front Microbiol. 2016 Dec 21;7:2080. doi: 10.3389/fmicb.2016.02080 (PMC5174107; doi:10.3389/fmicb.2016.02080)
Supplement: Supplementary file 1 [file Table2.PDF]

Supplementary Material

Comparative analysis of Type IV pilin in *Desulfuromonadales*

Chuanjun Shu [1]***, Ke Xiao*** [1]***, Qin Yan*** [1]***, and Xiao Sun*** [1*]

[1]*State Key Laboratory of Bioelectronics, School of Biological Science and Medical Engineering, Southeast University, Nanjing, China*

*** Correspondence:** *Xiao Sun, Email:* *xsun@seu.edu.cn*.

Supplementary Figures and Tables

Table S1. The detail information of complete genome sequence of eleven bacteria

	GC	Gene	Protein	Submit	Update
G．metallireducens GS-15	59.48%	3608	3520	2005/10/27	2015/2/4
G．sulfurreducens PCA	60.90%	3711	3430	2002/2/14	2014/12/22
G．uraniireducens Rf4	54.20%	4506	4417	2007/5/11	2015/2/4
G．lovleyi SZ	54.77%	3685	3614	2008/6/5	2015/2/4
G．bemidjiensis Bem	60.30%	4055	3942	2008/8/19	2015/2/4
G．sp. M21	60.50%	4143	4047	2009/7/7	2015/2/24
G．daltonii FRC-32	53.50%	3828	3750	2009/1/27	2015/2/4
G．sp. M18	61.20%	4522	4415	2011/1/27	2015/2/4
G．sulfurreducens KN400	61.00%	3610	3289	2010/6/17	2014/12/17
P. carbinolicus DSM 2380	55.10%	3386	3167	2012/9/13	2015/2/4
P. propionicus DSM 2379	59.00%	3598	3481	2006/10/31	2015/2/4

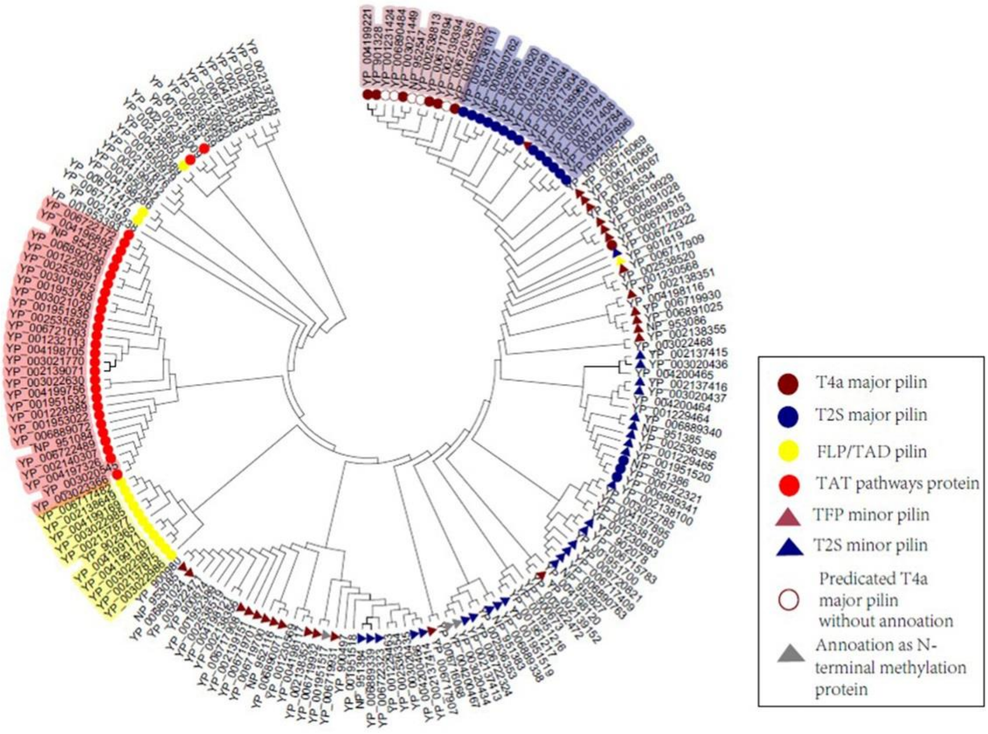

Figure S1. The cluster diagram of 183 predicted pilins

In this picture, there is a sign before pilin’s names means that it have corresponding annotation. Otherwise, the pilin may be have no annotation or is a false positive protein. The brown and blue shad areas cluster in one type. The brown shade area is T4a major pilins, blue shad area is T2S major pilins. The solid round in above shad areas means this pilin have annotation, however the hollow circle means no annotation. The yellow and red shade areas corresponding to non-TFP/T2S pilin.

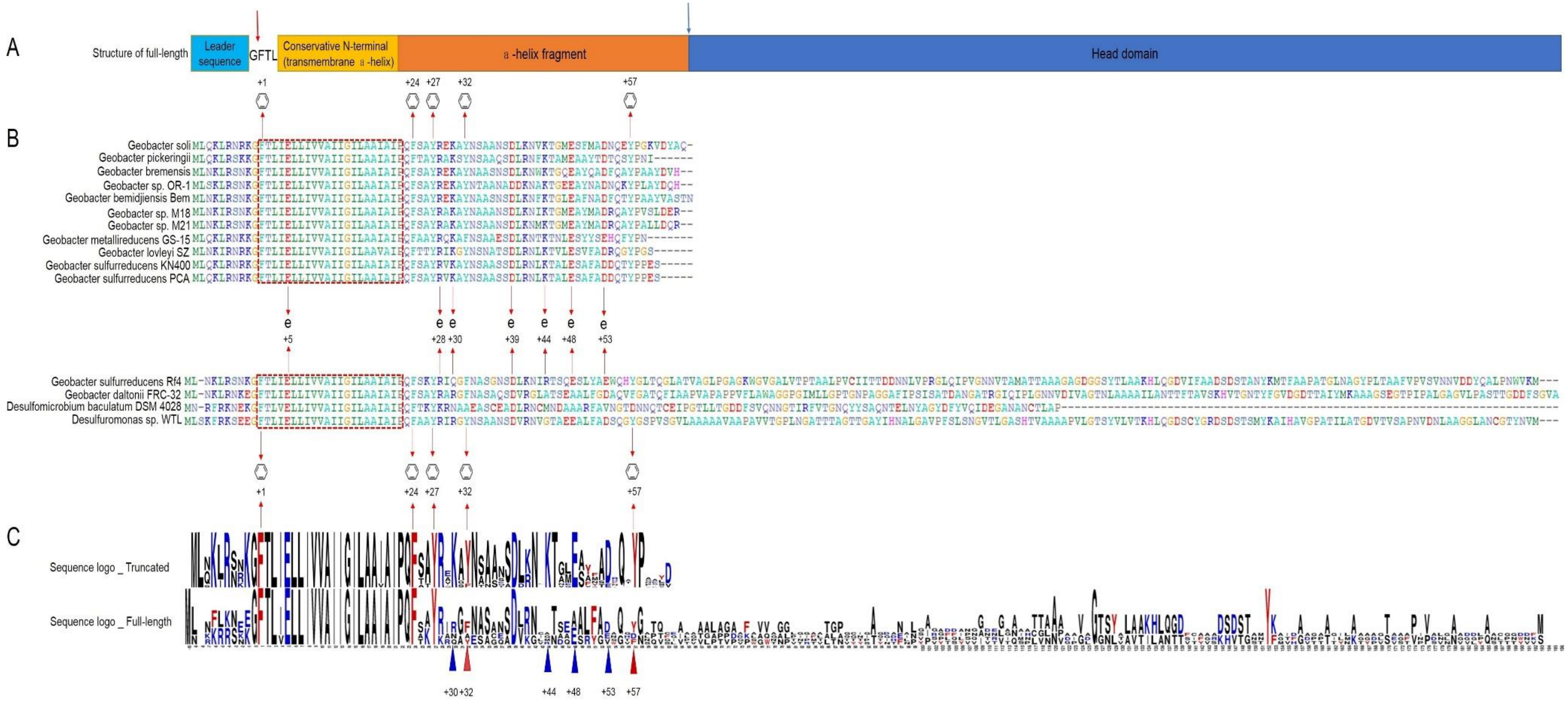

Figure S2. The conservative amino acids in GSU1496-like pilins and full-length pilins of Geobacter

A: The sequence construction of full-length pilin. The red arrow indicates the peptidase splice site, and the blue is the crack site of full-length change to truncate. B: The trans-membrane domain is shown in red dotted frame. ⬡ and **+** are employed for highlighting the sites of conserved aromatic amino acids and charged amino acids respectively. C: The sequence logo of truncated pilins (GSU1496-like) and full-length pilins in *Geobacter*. The red and blue triangles represent aromatic amino acids and charged amino acids respectively.

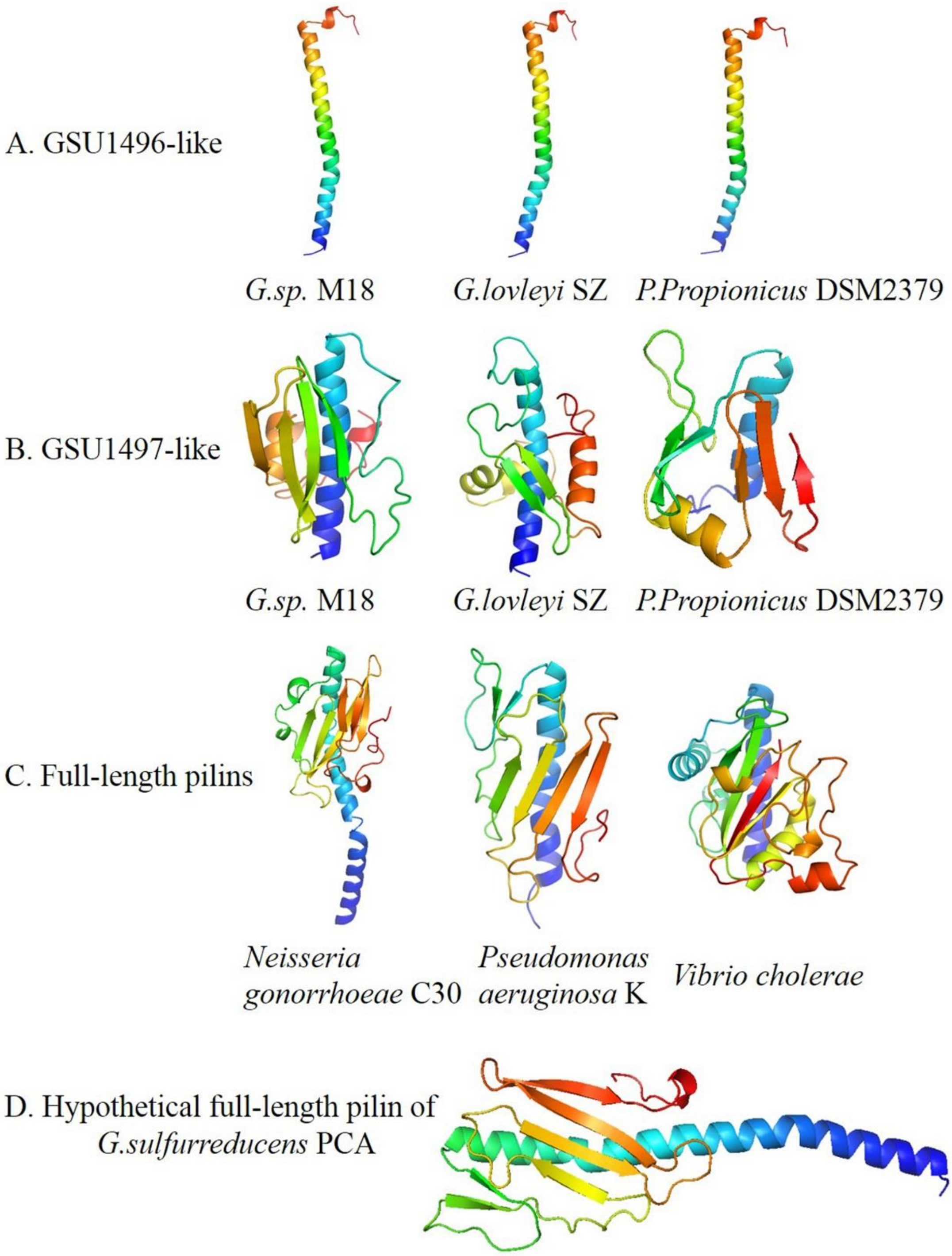

Figure S3. Structures of GSU1496-like pilins, GSU1497-like proteins, and full-length pilins

The structure of GSU1496-like pilin and GSU1497-like protein were predicted by ab initio calculations. The distribute of figureS3 as follows: The first line structures were GSU1496-like pilins, next were GSU1497-like proteins, and then were full-length pilins, last was hypothesis full-length pilin of PCA. The words were the brief name of bacteria that write in the corresponding structure directly below.

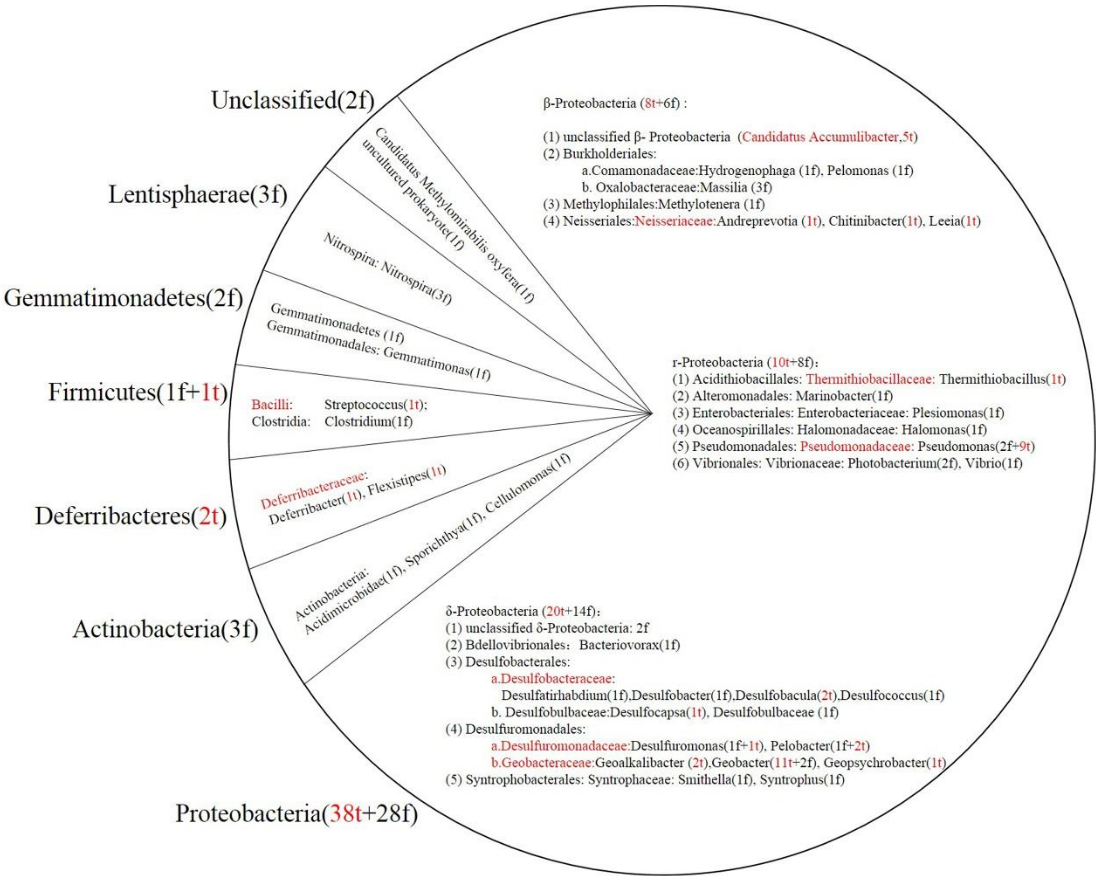

Figure S4. The species of bacterium for phylogenetic analysis

The names of phylum are marked on the outside of circle. The pie chart shows the detailed classification information. Numbers in brackets represent the strain distribution. In brackets, how many t mean how many truncated pilins, the number of f means that the number of full-length pilins. Red letters indicate the distribution of truncated pilins.

Table S2. The species of electricigens in Proteobacteria

	Class	Genus	Species	
Proteobacteria	α-Proteobacteria	Rhodopseudomonas	Rhodopseudomonas palustris	(1)
		Gluconobacter	Gluconobacter oxydans	(2)
		Ochrobactrum	Ochrobactrum anthropi	(3)
		Paracoccus	Paracoccus denitrificans	(4)
			Paracoccus pantotrophus	(5)
		Acidiphilium	Acidiphilium cryptum	(6)
		Rhizomicrobium	Rhizomicrobium electricum	(7)
	β-Proteobacteria	Rhodoferax	Rhodoferax ferrireducens	(8)
	γ-Proteobacteria	Shewanella	Shewanella putrefacions	(9)
			Shewanella marisflavi EP1	(10)
			Shewanella oneidensis	(11)
			Shewanella decolorationis	(12)
			Shewanella japonica	(13)
			Shewanella baltica	(14)
			Shewanella loihica PV-4	(15)
		Escherichia	Escherichia coli	(3)
		Enterobacter	Enterobacter cloacae	(16)
			Enterobacter aerogenes	(17)
		Aeromonas	Aeromonas hydrophila	(8)
		Pseudomonas	Pseudomonas alcaliphila	(18)
			Pseudomonas aeruginosa	(3)
		Proteus	Proteus vulgaris	(19)
		Citrobacter	Citrobacter sp SX-1	(20)
		Klebsiella	Klebsiella pneumoniae	(21)
		Erwinia	Erwinia dissolvens	(22)
	δ-Proteobacteria	Geobacter	Geobacter sulfurreducens	(23)
			Geobacter metallireducens	(24)
		Geopsychrobacter	Geopsychrobacter electrodophilus	(3)
		Desulfovibrio	Desulfovibrio desulfuricans	(3)
		Desulfuromonas	Desulfuromonas acetoxidans	(8)
		Desulfobulbus	Desulfobulbus propionicus	(25)
	ε-Proteobacteria	Arcobacter	Arcobacter butzleri	(26)

Table S3. The detail of truncated pilins in phylogenetic tree

Function	Genus/ species	Strains	Isolated from	Aerobism	Cluster	Class
phosphorus removal	Candidatus Accumulibacter sp. (27)	SK-02	wastewater	Anaerobic	2	β
		BA-94	wastewater	Anaerobic	2	β
		BA-92	wastewater	Anaerobic	2	β
		phosphatis	wastewater	Anaerobic	2	β
sulfate reducer	Desulfobacula	toluolica Tol2 (28)	marine sediment	Anaerobic	2	δ
		sp. TS(29)	oil-contaminated tidal flat	Anaerobic	3	δ
	Desulfocapsa sulfexigens	DSM 10523(30)	tidal flat	Anaerobic	3	δ
	Desulfuromonas sp.(31)	TF	marine sediments	Anaerobic	4	δ
		WTL	marine sediments	Anaerobic	4	δ
	Desulfomicrobium baculatum	DSM 4028(32)	water-saturated manganese carbonate ore	Anaerobic	4	δ
	Thermithiobacillus(33)	tepidarius	industrial anaerobic sludge pool	Anaerobic	2	γ
naphthalene degrader, crude oil hydrocarbons degrader	Pseudomonas(34)	stutzeri CCUG 29243	polluted marine sediments	facultative anaerobic	3	γ
		Stutzeri MF28	Mantle fluid	facultative anaerobic	3	γ
		stutzeri	polluted marine sediments	facultative anaerobic	3	γ
	Pseudomonas	sp. 10B238(35)	deep-sea sediment	Anaerobic	3	γ
		sp. ML96(36)		Aerobic	3	γ
		sp. BAY1663			3	γ
		mendocina(37)		Aerobic	3	γ
nitrogen oxides remover	Pseudomonas	mendocina DLHK(38)	Biotrickling reactor	Aerobic	3	γ

Degradation of the steroid compound cholate	Pseudomonas	sp. Chol1(39)	soil	facultative anaerobic	3	γ
Metal-reducing bacterium	Geobacter	metallireducens GS-15(40)	freshwater sediments of the Potomac River	Anaerobe	4	δ
		lovleyi SZ(41)	creek sediment	Anaerobe	4	δ
		pickeringii(42)	Sedimentary Kaolin Deposits	Anaerobe	4	δ
		sulfurreducens KN400(43)	a fuel cell	Anaerobe	4	δ
		sulfurreducens PCA(44)	surface sediments	Anaerobe	4	δ
		bremensis(45)	freshwater mud samples	Anaerobe	4	δ
		bemidjiensis Bem(46)	subsurface sediments	Anaerobe	4	δ
		sp. OR-1(47)	paddy soil	Anaerobe	4	δ
		soli(48)	forest soil	Anaerobe	4	δ
		sp. M21(49)	experiment	Anaerobic	4	δ
		sp. M18		Anaerobic	4	δ
	Pelobacter	seleniigenes(50)	wetland	Anaerobic	4	δ
		propionicus DSM 2379(51)	Marine muds	Anaerobic	4	δ
	Geoalkalibacter	ferrihydriticus DSM 17813(52)	bottom sediments of soda lakes	Anaerobic	4	δ
		subterraneus(53)	production water of oilfield	Anaerobic	4	δ
	Geopsychrobacter	Electrodiphilus (54)	Marine Sediment	Anaerobic	4	δ
	Andreprevotia chitinilytica(55)		forest soil	aerobic	2	β
	Leeia oryzae(56)		rice-field soil	aerobic	2	β
	Chitinibacter sp. ZOR0017(57)		soil	aerobic	2	β
	Streptococcus pneumoniae(58)		blood	facultative anaerobic	3	

sulfur-, nitrate- and arsenate-reducing thermophile	Deferribacter	Flexistipes sinusarabici DSM 4947(59)	sea water	anaerobic	4	
	Deferribacter	Deferribacter desulfuricans SSM1(60)	deep-sea	anaerobic	4	
Denitrification, phosphorus removal	Candidatus Accumulibacter sp.	SK-12(61)	wastewater	Anaerobic	5	β

Table S4. The detail of full-length pilins in phylogenetic tree

Function	Genus/ species	Strains	Isolated from	Aerobism	Lineage/cluster	Class
moderately halophilic	Halomonadaceae	Halomonas salina(62)	hypersaline soil	aerobic	L1	γ
	Oxalobacteraceae(63)	Massilia sp.Root335	Soil/air/water	aerobic	L1	β
		Massilia sp. JS1662	Soil/air/water	aerobic	L1	β
		Massilia sp. Root1485	Soil/air/water	aerobic	L1	β
degrade starch	Alteromonadaceae	Marinobacter salarius(64)	sea water	aerobic	L1	γ
denitrificans	Methylophilaceae	Methylotenera sp. L2L1(65)	lake	aerobic	L1	β
conditioned pathogen	Pseudomonas(66)	Pseudomonas aeruginosa CI27	widely distributed in nature	aerobic	L1	γ
		Pseudomonas aeruginosa C3719	widely distributed in nature	aerobic	L1	γ
Reduces Fe(III) Indirectly via Sulfide Production	Desulfuromonadales	Pelobacter carbinolicus DSM 2380(67, 68)	freshwater sediments, sewage sludge	anaerobic	L1	δ
Degradation of chlorobenzene	Enterobacteriaceae	Plesiomonas sp. ZOR0011(69)	freshwater	aerobic	L1	γ
bioluminescent microbe	Vibrionaceae	Photobacterium leiognathi(70)	warm tropical waters	facultative anaerobe	L1	γ
bioluminescent microbe	Vibrionaceae	Photobacterium angustum(71)	Sea Water	aerobic	L1	γ
Hydrogen-Oxidizing Bacteria	Comamonadadeae	Hydrogenophaga sp. Root209(72)	activated sludge	aerobic	L1	β
convert nitrite into nitrogen gas	Comamonadaceae	Pelomonas sp. Root1237(73)	water	aerobic	L1	β
acetate-oxidizing bacterium	Clostridiales	Clostridium homopropionicum DSM 5847	anoxic digested sludge	anaerobic(74)	L2/cluster 1	

unreported	Gemmatimonadetes	Gemmatirosa kalamazoonesis	soil(75)	aerobic	L2/cluster 1	
polyphosphate-accumulating micro-organism	Gemmatimonadetes(76)	Gemmatimonas sp. SG8 28	soil	aerobic	L2/cluster 1	
Nitrite oxidation into nitrates	Nitrospiraceae	Nitrospira defluvii(77)	marine recirculation aquaculture system/water	aerobic	L2/cluster 1	
iron-oxidizing	Actinobacteria	Ferrimicrobium acidiphilum DSM 19497	Acid mine drainage waters	aerobic(78)	L2/cluster 1	
unreported	Sporichthyaceae	Sporichthya polymorpha	soil(79)	facultative anaerobic	L2/cluster 1	
cellulose-degrading	Cellulomonadaceae	Cellulomonas sp. FA1(80)	plant material	facultative anaerobic	L2/cluster 1	
biocontrol agent	Bacteriovoracaceae	Bacteriovorax sp. Seq25 V	Marine/salt-water	aerobic(81)	L2/cluster 1	δ
butyrate-oxidizing, sulfate-reducing	Desulfobacteraceae	Desulfatirhabdium butyrativorans(82)	anaerobic bioreactor	anaerobic	L2/cluster 1	δ
sulfate-reducing	Desulfobacteraceae	Desulfococcus multivorans DSM 2059	soil	anaerobic(83)	L2/cluster 1	δ
Nitrite oxidation into nitrates	Nitrospiraceae	Nitrospira moscoviensis	water(84)	aerobic	L2/cluster 1	
		Nitrospira sp. 2	water(85)	aerobic	L2/cluster 1	
sulfate reducer	Desulfobacteraceae	Desulfobacter postgatei 2ac9	brackish water	anaerobe(86)	L2/cluster 2	δ
oxidation of sulfide to sulfate	proteobacterium	delta proteobacterium MLMS-1	Mono Lake(87)	anaerobe	L2/cluster 2	δ
Sulfidogenic activity	Deltaproteobacteria	Desulfurivibrio alkaliphilus AHT 2	Natrun lakes(88)	anaerobe	L2/cluster 2	δ
unknown	Deltaproteobacteria	Deltaproteobacteria bacterium SM23 61	unknown	anaerobic	L2/cluster 2	δ
hydrogen-using microorganism	syntrophaceae	Syntrophus aciditrophicus SB(89)	sewage treatment plant	anaerobic	L2/cluster 2	δ

unknown	syntrophaceae	Smithella sp. F21	unknown	anaerobic(90)	L2/cluster 2	δ
sulfate-reducing	Desulfuromonas	Desulfuromonas sp. WTL(91)	marine sediments	anaerobic	L2/cluster 4	δ
Metal-reducing bacterium	Geobacter	Geobacter uraniireducens Rf4(92)	subsurface sediment	anaerobic	L2/cluster 4	δ
sulfur-reducing	Desulfomicrobiaceae	Desulfomicrobium baculatum DSM 4028(93)	water-saturated manganese carbonate ore	anaerobic	L2/cluster 4	δ
Metal-reducing bacterium	Geobacter	Geobacter daltonii FRC-32(94)	contaminated subsurface	anaerobic	L2/cluster 4	δ
Methanotroph; nitrite-reducing Denitrification	unclassified	Candidatus Methylomirabilis oxyfera(95)	freshwater	anaerobic	L2/cluster 5	

Table S5. The homology gene of GSU1497 predicted by PSI-BLAST

	gene	length	score	consistency%	similarity%	E-values
G.sulfurreducens PCA	GSU1497	124	357	100.0	100.0	7.0e-32
G.sulfurreducens KN400	KN400_3442	124	357	100.0	100.0	7.0e-32
G.sp. M18	GM18_2491	120	280	20.0	40.0	6.0e-23
G.bemidjiensis Bem	Gbem_2589	136	270	21.0	42.0	8.0e-22
G.sp.M21	GM21_1637	117	268	18.0	39.0	1.0e-21
G.lovleyi SZ	Glov_2095	119	236	20.0	45.0	7.0e-18
G.metallireducens GS-15	Gmet_1400	113	232	27.0	43.0	2.0e-17
P.propionicus DSM 2379	Ppro_1657	86	201	19.0	43.0	8.0e-14

Table S6. Locus tags and location of GSU1496-like pilins and GSU1497-like proteins

	GSU1496-like	GSU1497 -like
G.sulfurreducens.PCA	GSU1496 (1642254-1642526)*	GSU1497 (1642554-1642928)
G.sulfurreducens.KN400	KN400_1523 (1611908-1612180)	KN400_3442 (1612208-1612582)
G.sp. M21	GM21_1636 (1906956-1907180)	GM21_1637 (1907215-1907568)
G.sp. M18	GM18_2492 (2983576-2983800)	GM18_2491 (2983182-2983544)
G.bemidjiensis Bem	Gbem_2590 (2999491-2999721)	Gbem_2589 (2999067-2999477)
G.metallireducens GS-15	Gmet_1399 (1568489-1568698)	Gmet_1400 (1568751-1569092)
G.lovleyi SZ	Glov_2096 (2234569-2234781)	Glov_2095 (2234166-2234525)
P.propionicus DSM 2379	Ppro_1656 (1775631-1775855)	Ppro_1657 (1775997-1776257)

*The value in bracket represent the location in genome of nucleotide acid that encoding GSU1496-like pilin or GSU1497-like protein.

References:

1. Lovley, D.R., The microbe electric: conversion of organic matter to electricity. Curr Opin Biotechnol, 2008. 19(6): p. 564-71.

2. Çinar, M.G.Y.U., Mikrobiyal Yakıt Hücrelerinde Ekstraselüler Elektron Transferleri, in Biyoloji Bilimleri Araştırma Dergisi. 2011. p. 81-85.

3. Logan, B.E., Exoelectrogenic bacteria that power microbial fuel cells. NATURE REVIEWS MICROBIOLOGY, 2009. 7(5): p. 375-381.

4. Mahidhara, G. and V.R. Chintalapati, Eco-physiological and interdisciplinary approaches for empowering biobatteries. Annals of Microbiology, 2015.

5. Rabaey, K., et al., Microbial fuel cells for sulfide removal. Environ Sci Technol, 2006. 40(17): p. 5218-24.

6. San, M.P., et al., Draft genome sequence of the electricigen Acidiphilium sp. strain PM (DSM 24941). J Bacteriol, 2011. 193(19): p. 5585-6.
7. Kodama, Y. and K. Watanabe, Rhizomicrobium electricum sp. nov., a facultatively anaerobic, fermentative, prosthecate bacterium isolated from a cellulose-fed microbial fuel cell. Int J Syst Evol Microbiol, 2011. 61(Pt 8): p. 1781-5.
8. Lovley, D.R., Bug juice: harvesting electricity with microorganisms. Nat Rev Microbiol, 2006. 4(7): p. 497-508.
9. Myers, C.R. and K.H. Nealson, Respiration-linked proton translocation coupled to anaerobic reduction of manganese (IV) and iron (III) in Shewanella putrefaciens MR-1. J Bacteriol, 1990. 172(11): p. 6232-8.
10. Ning, G., Reductive Biotransformation of Pendimethalin by Shewanella Marisflavi EP1 under Anaerobic Conditions. Environmental science & technology, 2015. 2(0013-936X): p. 005.
11. Lanthier, M., K.B. Gregory and D.R. Lovley, Growth with high planktonic biomass in Shewanella oneidensis fuel cells. FEMS Microbiol Lett, 2008. 278(1): p. 29-35.
12. Sikora, A.J.W.K. and R.A.S.P. Aleksandra Chojnacka, Selection of Bacteria Capable of Dissimilatory Reduction of Fe(III) from a Long-term Continuous Culture on Molasses and Their Use in a Microbial Fuel Cell, in J. Microbiol. Biotechnol. 2011. p. 305-316.
13. Hou, B., J. Sun and Y. Hu, Effect of enrichment procedures on performance and microbial diversity of microbial fuel cell for Congo red decolorization and electricity generation. Appl Microbiol Biotechnol, 2011. 90(4): p. 1563-72.
14. Liang, P., et al., [Influence of environmental factors on electricity production by microbial fuel cell inoculation Shewanella baltica]. Huan Jing Ke Xue, 2009. 30(7): p. 2148-52.
15. Wenguo Wu, L.B.X.L., Nanograss array boron-doped diamond electrode for enhanced electron transfer from Shewanella loihica PV-4. Electrochemistry Communications, 2011. doi: 10.1016/j.elecom.2011.05.025.
16. Li, Y., et al., Spontaneous arsenic (III) oxidation with bioelectricity generation in single-chamber microbial fuel cells. J Hazard Mater, 2015. 306: p. 8-12.
17. Zhang, J.T., et al., [Mechanisms of bioelectricity generation in Enterobacter aerogenes-based microbial fuel cells]. Huan Jing Ke Xue, 2009. 30(4): p. 1215-20.
18. Lu, L., et al., Graphene oxide and H2 production from bioelectrochemical graphite oxidation. Sci Rep, 2015. 5: p. 16242.
19. Parida, D., A. Yadav and A. Muralidharan, Consortium Building For PEM MFC Using Synthetic Media As Substrate. Nature Precedings, 2011. hdl:10101/npre.2011.6632.1.
20. Huang, J., et al., Exoelectrogenic bacterium phylogenetically related to Citrobacter freundii, isolated from anodic biofilm of a microbial fuel cell. Appl Biochem Biotechnol, 2015. 175(4): p. 1879-91.
21. Zhan, Y., et al., Analysis of Microbe in Microbial Fuel Cell. Journal of Chemical Engineering of Chinese Universities, 2009. 3: p. 016.
22. Reddy, L.V., S.P. Kumar and Y. Wee, Microbial Fuel Cells (MFCs)-a novel source of energy for new millennium. Current Research Technology and Education Topics in Applied Microbiology and Microbial Biotechnology, 2010. 2(13): p. 956-964.
23. Liu, X., et al., A Geobacter sulfurreducens strain expressing pseudomonas aeruginosa type IV pili localizes OmcS on pili but is deficient in Fe(III) oxide reduction and current production. Appl Environ Microbiol, 2014. 80(3): p. 1219-24.
24. Tremblay, P.L., et al., A genetic system for Geobacter metallireducens: role of the flagellin and pilin

in the reduction of Fe(III) oxide. ENVIRONMENTAL MICROBIOLOGY REPORTS, 2012. 4(1SI): p. 82-88.
25. Gong, Y., et al., Sulfide-driven microbial electrosynthesis. Environmental science & technology, 2012. 47(1): p. 568-573.
26. Sikora, A., et al., Selection of bacteria capable of dissimilatory reduction of Fe (III) from a long-term continous culture on molasses and their use in a microbial fuel cell. Journal of microbiology and biotechnology, 2011. 21(3): p. 305-316.
27. Kim, J.M., et al., Analysis of the fine-scale population structure of "Candidatus accumulibacter phosphatis" in enhanced biological phosphorus removal sludge, using fluorescence in situ hybridization and flow cytometric sorting. Appl Environ Microbiol, 2010. 76(12): p. 3825-35.
28. Wohlbrand, L., et al., Complete genome, catabolic sub-proteomes and key-metabolites of Desulfobacula toluolica Tol2, a marine, aromatic compound-degrading, sulfate-reducing bacterium. Environ Microbiol, 2013. 15(5): p. 1334-55.
29. Kim, S.J., et al., Draft genome sequence of an aromatic compound-degrading bacterium, Desulfobacula sp. TS, belonging to the Deltaproteobacteria. FEMS Microbiol Lett, 2014. 360(1): p. 9-12.
30. Finster, K.W., et al., Complete genome sequence of Desulfocapsa sulfexigens, a marine deltaproteobacterium specialized in disproportionating inorganic sulfur compounds. Stand Genomic Sci, 2013. 8(1): p. 58-68.
31. Zhang, T., et al., Sulfur oxidation to sulfate coupled with electron transfer to electrodes by Desulfuromonas strain TZ1. Microbiology, 2014. 160(Pt 1): p. 123-129.
32. Copeland, A., et al., Complete genome sequence of Desulfomicrobium baculatum type strain (X). Stand Genomic Sci, 2009. 1(1): p. 29-37.
33. Yang, H., et al., Isolation of sulfide remover strain Thermithiobacillus tepidarius JNU-2, and scale-up bioreaction for sulfur regeneration. Annals of Microbiology, 2015. 65(1): p. 553-563.
34. Brunet-Galmes, I., et al., Complete genome sequence of the naphthalene-degrading bacterium Pseudomonas stutzeri AN10 (CCUG 29243). J Bacteriol, 2012. 194(23): p. 6642-3.
35. Pan, H.Q. and J.C. Hu, Draft genome sequence of the novel strain Pseudomonas sp. 10B238 with potential ability to produce antibiotics from deep-sea sediment. Mar Genomics, 2015. 23: p. 55-7.
36. Li, X., J. Blom and Y. Zeng, Whole genome sequences of a free-living Pseudomonas sp. strain ML96 isolated from a freshwater Maar Lake. Mar Genomics, 2015. 24 Pt 3: p. 219-21.
37. Aragone, M.R., et al., Pseudomonas mendocina, an environmental bacterium isolated from a patient with human infective endocarditis. J Clin Microbiol, 1992. 30(6): p. 1583-4.
38. Wong, C.F., et al., Genome sequence of Pseudomonas mendocina DLHK, isolated from a biotrickling reactor. J Bacteriol, 2012. 194(22): p. 6326.
39. Holert, J., et al., Genome Sequence of Pseudomonas sp. Strain Chol1, a Model Organism for the Degradation of Bile Salts and Other Steroid Compounds. Genome Announc, 2013. 1(1).
40. Lovley, D.R., et al., Geobacter metallireducens gen. nov. sp. nov., a microorganism capable of coupling the complete oxidation of organic compounds to the reduction of iron and other metals. Arch Microbiol, 1993. 159(4): p. 336-44.
41. Sung, Y., et al., Geobacter lovleyi sp. nov. strain SZ, a novel metal-reducing and tetrachloroethene-dechlorinating bacterium. Appl Environ Microbiol, 2006. 72(4): p. 2775-82.
42. Badalamenti, J.P. and D.R. Bond, Complete Genome of Geobacter pickeringii G13T, a Metal-Reducing Isolate from Sedimentary Kaolin Deposits. Genome Announc, 2015. 3(2).

43. Butler, J.E., et al., Comparative genomic analysis of Geobacter sulfurreducens KN400, a strain with enhanced capacity for extracellular electron transfer and electricity production. BMC genomics, 2012. 13(1): p. 471.
44. Selvaraj, A., et al., A Whole Genome Pairwise Comparative and Functional Analysis of Geobacter sulfurreducens PCA. Isrn Computational Biology, 2013. 2013.
45. Straub, K.L. and B.E. Buchholz-Cleven, Geobacter bremensis sp. nov. and Geobacter pelophilus sp. nov., two dissimilatory ferric-iron-reducing bacteria. Int J Syst Evol Microbiol, 2001. 51(Pt 5): p. 1805-8.
46. Nevin, K.P., et al., Geobacter bemidjiensis sp. nov. and Geobacter psychrophilus sp. nov., two novel Fe(III)-reducing subsurface isolates. Int J Syst Evol Microbiol, 2005. 55(Pt 4): p. 1667-74.
47. Ohtsuka, T., et al., Arsenic dissolution from Japanese paddy soil by a dissimilatory arsenate-reducing bacterium Geobacter sp. OR-1. Environ Sci Technol, 2013. 47(12): p. 6263-71.
48. Zhou, S., et al., Geobacter soli sp. nov., a dissimilatory Fe (III)-reducing bacterium isolated from forest soil. Int J Syst Evol Microbiol, 2014. 64(Pt 11): p. 3786-91.
49. Emtiazi, G., Comparative modelling of 3D-structure of Geobacter sp. M21 (a metal reducing bacteria) Mn-Fe superoxide dismutase and its binding pr..., in Iranian Journal of Environmental Technology. 2014. p. 19-30.
50. Narasingarao, P. and M.M. Haggblom, Pelobacter seleniigenes sp. nov., a selenate-respiring bacterium. Int J Syst Evol Microbiol, 2007. 57(Pt 9): p. 1937-42.
51. Sehink, B., Fermentation of 2,3-butanediol by Pelobacter carbinolicus sp. nov. and Pelobacter propionicus sp. nov., and evidence for propionate formation from C2 compounds, in Arch Microbiol. 1984. p. 33-41.
52. Zavarzina, D.G., et al., [Geoalkalibacter ferrihydriticus gen. nov., sp. nov., the first alkaliphilic representative of the family Geobacteraceae, isolated from a soda lake]. Mikrobiologiia, 2006. 75(6): p. 775-85.
53. Greene, A.C., B.K. Patel and S. Yacob, Geoalkalibacter subterraneus sp. nov., an anaerobic Fe(III)- and Mn(IV)-reducing bacterium from a petroleum reservoir, and emended descriptions of the family Desulfuromonadaceae and the genus Geoalkalibacter. Int J Syst Evol Microbiol, 2009. 59(Pt 4): p. 781-5.
54. Holmes, D.E., et al., Potential role of a novel psychrotolerant member of the family Geobacteraceae, Geopsychrobacter electrodiphilus gen. nov., sp. nov., in electricity production by a marine sediment fuel cell. Appl Environ Microbiol, 2004. 70(10): p. 6023-30.
55. Weon, H.Y., et al., Andreprevotia chitinilytica gen. nov., sp. nov., isolated from forest soil from Halla Mountain, Jeju Island, Korea. Int J Syst Evol Microbiol, 2007. 57(Pt 7): p. 1572-5.
56. Lim, J.M., et al., Leeia oryzae gen. nov., sp. nov., isolated from a rice field in Korea. Int J Syst Evol Microbiol, 2007. 57(Pt 6): p. 1204-8.
57. Gao, C., et al., Characterization of extracellular chitinase from Chitinibacter sp. GC72 and its application in GlcNAc production from crayfish shell enzymatic degradation. Biochemical Engineering Journal, 2015. 97: p. 59-64.
58. Tettelin, H., et al., Complete genome sequence of a virulent isolate of Streptococcus pneumoniae. Science, 2001. 293(5529): p. 498-506.
59. Fiala, G., et al., Stetter Flexistipes sinusarabici, a novel genus and species of eubacteria occurring in the Atlantis II Deep brines of the Red Sea. Archives of Microbiology, 1990. 154(2): p. 120-126.
60. Takai, K., H. Kobayashi and K.K. Nealson, Deferribacter desulfuricans sp nov., a novel sulfur-,

nitrate- and arsenate-reducing thermophile isolated from a deep-sea hydrothermal vent. International Journal of Systematic & Evolutionary Microbiology, 2003. 53(3): p. 839-846.
61. Flowers, J.J., et al., Denitrification capabilities of two biological phosphorus removal sludges dominated by different "Candidatus Accumulibacter" clades. Environ Microbiol Rep, 2009. 1(6): p. 583-588.
62. Valderrama, M.J., et al., Influence of salt concentration on the cellular fatty acid composition of the moderately halophilic bacterium Halomonas salina. Res Microbiol, 1998. 149(9): p. 675-9.
63. Maya, O., H. Yitzhak and M. Dror, Ecology of root colonizing Massilia (Oxalobacteraceae). Plos One, 2012. 7(7): p. e40117.
64. Hooi Jun, N., et al., Marinobacter salarius sp. nov. and Marinobacter similis sp. nov., isolated from sea water. Plos One, 2014. 9(9): p. e106514-e106514.
65. McTaggart, T.L., et al., Draft genome sequences of five new strains of methylophilaceae isolated from lake washington sediment. Genome Announc, 2015. 3(1).
66. Evans, D.J., et al., Susceptibility of Pseudomonas aeruginosa and Escherichia coli biofilms towards ciprotoxin: effect of specific growth rate. Journal of Antimicrobial Chemotherapy, 1991. 27.
67. Schink, B., Fermentation of 2,3-butanediol by Pelobacter carbinolicus sp. nov. and Pelobacter propionicus sp. nov., and evidence for propionate formation from C 2 compounds. Archives of Microbiology, 1984. 137(1): p. 33-41.
68. Haveman, S., et al., Genome-wide gene expression patterns and growth requirements suggest that Pelobacter carbinolicus reduces Fe(III) indirectly via sulfide production. Applied & Environmental Microbiology, 2008. 74(14): p.: 4277 – 4284.
69. Luo, R., N.A. University and N.Z. Suqin, Degradation characteristics of chlorobenzene by Plesiomonas sp. L1 . China Environmentalence, 1998. 18: p. 272-275.
70. Dunn, A.K., et al., Regulation of Bioluminescence in Photobacterium leiognathi Strain KNH6. Journal of Bacteriology, 2015. 197(23): p. 3676-85.
71. Sabine, M.S., et al., Proteome analysis of the UVB-resistant marine bacterium Photobacterium angustum S14. Plos One, 2012. 7(8): p.: e42299.
72. Hoven, R.N.V. and J.M. Santini, Arsenite oxidation by the heterotroph Hydrogenophaga sp. str. NT-14: the arsenite oxidase and its physiological electron acceptor. Biochimica Et Biophysica Acta, 2004. 1656(2-3): p. 148 – 155.
73. Adav, S.S., D.J. Lee and J.Y. Lai, Microbial community of acetate utilizing denitrifiers in aerobic granules. Appl Microbiol Biotechnol, 2010. 85(3): p. 753-62.
74. Beck, M.H., et al., Draft Genome Sequence of the Strict Anaerobe Clostridium homopropionicum LuHBu1 (DSM 5847). Genome Announcements, 2015. 3(5).
75. Debruyn, J.M., et al., Gemmatirosa kalamazoonesis gen. nov., sp. nov., a member of the rarely-cultivated bacterial phylum Gemmatimonadetes. Journal of General & Applied Microbiology, 2013. 59(4): p. 305-12.
76. Zhang, H., et al., Gemmatimonas aurantiaca gen. nov., sp nov., a gram-negative, aerobic, polyphosphate-accumulating micro-organism, the first cultured representative of the new bacterial phylum Gemmatimonadetes phyl. nov. International Journal of Systematic & Evolutionary Microbiology, 2003. 53(Pt 4): p. 1155-1163.
77. Frank, M., et al., Environmental genomics reveals a functional chlorite dismutase in the nitrite-oxidizing bacterium 'Candidatus Nitrospira defluvii'. Environmental Microbiology, 2008. 10(11): p. 3043 – 3056.

78. Johnson, D.B., et al., Ferrimicrobium acidiphilum gen. nov., sp. nov. and Ferrithrix thermotolerans gen. nov., sp. nov.: heterotrophic, iron-oxidizing, extremely acidophilic actinobacteria. International Journal of Systematic & Evolutionary Microbiology, 2009. 59(Pt 5): p. 1082-1089.
79. Rainey, F.A., et al., Sporichthya polymorpha represents a novel line of descent within the order Actinomycetales. Fems Microbiology Letters, 1993. 109(2-3): p. 263 – 267.
80. Cohen, M.F., et al., Genome Sequence of the Alkaline-Tolerant Cellulomonas sp. Strain FA1. Genome Announcements, 2015. 3(3).
81. Wen, C., et al., Evaluating the potential of marine Bacteriovorax sp. DA5 as a biocontrol agent against vibriosis in Litopenaeus vannamei larvae. Veterinary Microbiology, 2014. 173(1-2): p. 84-91.
82. Balk, M., et al., Desulfatirhabdium butyrativorans gen. nov., sp. nov., a butyrate-oxidizing, sulfate-reducing bacterium isolated from an anaerobic bioreactor. International Journal of Systematic & Evolutionary Microbiology, 2008. 58(1): p. 110-115.
83. Gorontzy, T., J. Küver and K.H. Blotevogel, Microbial transformation of nitroaromatic compounds under anaerobic conditions. Journal of General Microbiology, 1993. 139 Pt 6(6): p. 1331-1336.
84. Ehrich, S., et al., A new obligately chemolithoautotrophic, nitrite-oxidizing bacterium,Nitrospira moscoviensis sp. nov. and its phylogenetic relationship. Archives of Microbiology, 1995. 164(1): p. 16-23.
85. Andreas, S., et al., Microenvironments and distribution of nitrifying bacteria in a membrane-bound biofilm. Environmental Microbiology, 2008. 2(6): p. 680-686.
86. Ingvorsen, K., A.J. Zehnder and B.B. J Rgensen, Kinetics of Sulfate and Acetate Uptake by Desulfobacter postgatei. Applied & Environmental Microbiology, 1984. 47(2): p. 403-8.
87. Planer-Friedrich, B., et al., Anaerobic Chemolithotrophic Growth of the Haloalkaliphilic Bacterium Strain MLMS-1 by Disproportionation of Monothioarsenate. Environmental Science & Technology, 2015. 49(11): p. 6554-63.
88. Sorokin, D.Y., et al., Dethiobacter alkaliphilus gen. nov. sp. nov., and Desulfurivibrio alkaliphilus gen. nov. sp. nov.: two novel representatives of reductive sulfur cycle from soda lakes. Extremophiles, 2008. 12(3): p. 431-9.
89. Elshahed, M.S., et al., Metabolism of benzoate, cyclohex-1-ene carboxylate, and cyclohexane carboxylate by "Syntrophus aciditrophicus" strain SB in syntrophic association with H(2)-using microorganisms. Applied & Environmental Microbiology, 2001. 67(4): p. 1728-38.
90. Boonfei, T., et al., Draft Genome Sequences of Three Smithella spp. Obtained from a Methanogenic Alkane-Degrading Culture and Oil Field Produced Water. Genome Announcements, 2014. 2(5).
91. Pfennig, N. and H. Biebl, Desulfuromonas acetoxidans gen. nov. and sp. nov., a new anaerobic, sulfur-reducing, acetate-oxidizing bacterium. Archives of Microbiology, 1976. 110(1): p. 3-12.
92. Shelobolina, E.S., et al., Geobacter uraniireducens sp. nov., isolated from subsurface sediment undergoing uranium bioremediation. Int J Syst Evol Microbiol, 2008. 58(Pt 5): p. 1075-8.
93. Alex, C., et al., Complete genome sequence of Desulfomicrobium baculatum type strain (X). Standards in Genomic Sciences, 2008. 1(3): p. 242-252.
94. Prakash, O., et al., Geobacter daltonii sp. nov., an Fe(III)- and uranium(VI)-reducing bacterium isolated from a shallow subsurface exposed to mixed heavy metal and hydrocarbon contamination. Int J Syst Evol Microbiol, 2010. 60(Pt 3): p. 546-53.
95. Wu, M.L., et al., A new intra-aerobic metabolism in the nitrite-dependent anaerobic methane-oxidizing bacterium Candidatus 'Methylomirabilis oxyfera'. Biochem Soc Trans, 2011. 39(1): p. 243-8.
